# Alternative extracellular disulfide bond formation by N-terminal cysteines supports functional expression of odorant receptors

**DOI:** 10.1016/j.jbc.2026.113319

**Published:** 2026-07-13

**Authors:** Nonoko Muto, Tomoyo Koshizawa, Miki Takeda, Xiaoyang Serene Hu, Ryosuke Inoue, Masafumi Yohda, Hiroaki Matsunami, Yosuke Fukutani

**Affiliations:** 1 Department of Biotechnology and Life Science, Tokyo University of Agriculture and Technology, Koganei, Tokyo, Japan; 2 Department of Molecular Genetics and Microbiology, Duke University School of Medicine, Durham, North Carolina, USA; 3 Department of Advanced Interdisciplinary Science, Tokyo University of Agriculture and Technology, Koganei, Tokyo, Japan

**Keywords:** disulfide bond, G-protein coupled receptor, membrane protein, odorant receptor, protein folding

## Abstract

Mammalian odorant receptors (ORs), a large family of G protein-coupled receptors, rely on conserved intramolecular disulfide bonds for proper folding and their function. However, some ORs lack the typical cysteine in extracellular loop 2, raising questions regarding alternative structural-stabilization mechanisms. Here, we identified and characterized an unconventional disulfide bond between a unique N-terminal cysteine and extracellular loop 2 cysteine in OR51E2, the first mammalian OR with an experimentally resolved structure. Using mutagenesis and functional assays in heterologous cells, we demonstrated that this N-terminal cysteine is essential for receptor surface expression and ligand responsiveness. Furthermore, AlphaFold structural predictions correlated with experimental data, indicating that alternative disulfide bonds contribute to receptor stability. These findings reveal a flexible disulfide bonding strategy in ORs, suggesting that alternative extracellular cysteine linkages can compensate for missing conserved bonds, thereby supporting receptor folding and functional diversification within the G protein-coupled receptor family.

Olfaction, a fundamental sensory system in animals, plays a crucial role in detecting and interpreting environmental changes, thereby enabling navigation through daily life. Mammalian odorant receptors (ORs), members of the largest family of G protein-coupled receptors (GPCRs), comprise approximately 400 and 1100 members in humans and mice, respectively (1, 2). These receptors are expressed on the cilia of olfactory sensory neurons (3) and are essential for detecting and discriminating a wide variety of odors (4, 5). In addition to mediating odor detection, a subset of ORs, including OR51E2 and its close paralog OR51E1, which respond to short-chain fatty acids, are expressed in extranasal tissues, where they participate in the physiological regulation of the gut and kidney and in the pathophysiology of prostate cancer (6, 7, 8).

As class A GPCRs, ORs exhibit a characteristic structure, including seven transmembrane domains (TMs), an extracellular N terminus, an intracellular C terminus, and three extracellular loops (ECLs) interconnected by three intracellular loops. Intramolecular disulfide bonds are crucial for the proper three-dimensional structure of many proteins, including GPCRs. In class A GPCRs, a conserved disulfide bond between the upper end of TM3 (Ballesteros-Weinstein position (9) BW3.25) and ECL2 (BW45.50) ensures correct structural formation (10, 11). In addition, in ORs, two conserved cysteines in ECL2 (BW45.40 and BW45.60) form a second disulfide bond that stabilizes the three-dimensional structure of the receptor, which is critical for ligand recognition and activation (12). ECL2 has been implicated in ligand binding to ORs (13). Cryo-EM analysis of OR51E2, the first and only native OR with an experimental structure, in its agonist-bound state revealed that ECL2 forms a "lid" over the ligand-binding pocket. Furthermore, the residue Q181 (BW45.53) within ECL2 plays a crucial role in recognizing fatty acid ligands, suggesting that the stereocoordination of ECL2 affects OR ligand recognition (14). The N-terminal and TM1 regions also contribute to this "lid" structure, which covers the upper portion of the receptor and is enclosed by ECL1 and ECL2 (14).

In structural studies of many GPCRs, the N-terminal region is often disordered without affecting the core structure or is often deleted or modified for protein production (15). For example, in class A GPCRs, such as adrenergic receptor-beta 2, the N-terminal region does not typically interact with other domains (16). However, in the cryo-EM structures of trace amine-associated receptor 9 (TAAR9), another class A GPCR, a disulfide bond was identified between cysteines in the N-terminal region and ECL2 (17). Mutating these cysteines significantly reduced ligand responsiveness, indicating that this disulfide bond is essential for ligand recognition (17). However, the mechanism by which the disulfide bond between the N-terminal domain and an ECL domain contributes to the function of broader GPCRs remains unclear.

In this study, we investigated the unique N-terminal structure and intramolecular disulfide bonds in ORs to understand their roles in structural formation and functional expression. Our analysis was motivated by the Cryo-EM structure of OR51E2, which possesses a cysteine in its N-terminal region that likely forms a disulfide bond with ECL2. To explore the functional significance of this N-terminal cysteine, we compared OR51E2 with its close homolog, OR51E1, which lacks an N-terminal cysteine (14). Our findings demonstrate the importance and flexibility of disulfide bonds within the OR family, as they influence the functional expression of ORs. Our study suggests that some GPCRs rely on N-terminal cysteines to form intramolecular disulfide bonds, which contribute to their structural integrity and function.

## Results

### Prediction of intramolecular disulfide bond involving N-terminal cysteine in human class A GPCRs

#### Occurrence of N-terminal cysteines and predicted disulfide bonds

To explore whether N-terminal cysteine residues form intramolecular disulfide bonds with the extracellular domains of class A GPCRs, we first conducted an *in silico* survey using available sequence and structural data. Since OR51E2 is currently the only native OR with an experimentally determined structure, we initially focused on non-OR GPCRs, for which many high-resolution structures have been determined. This allowed us to evaluate the reliability of the structural predictions before focusing on ORs.

**Figure 1:**
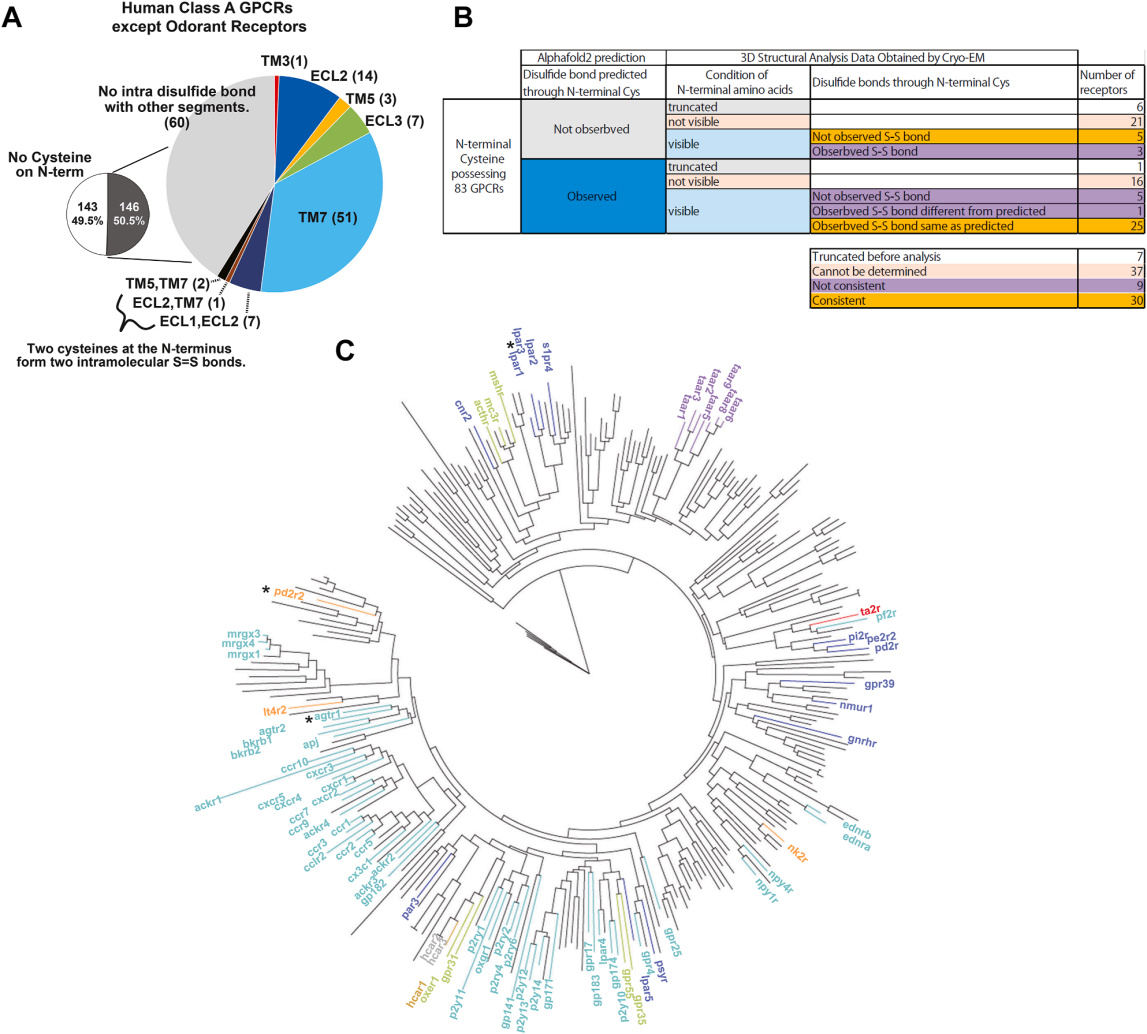
**Class A GPCRs, excluding ORs, with cysteine residues in the N-terminal region.***A*, among the 289 class A GPCRs, 146 receptors possess cysteine residues in their N-terminal regions. Of these, 60 receptors (*gray*) do not form disulfide bonds between the N terminus and other domains of the protein. The remaining 86 receptors form intramolecular disulfide bonds involving N-terminal cysteines and cysteines in other domains. These include one receptor (*red*) with a corresponding cysteine in transmembrane domain 3 (TM3), 14 receptors (*blue*) with cysteines in extracellular loop 2 (ECL2), three receptors (*orange*) with cysteines in TM5, seven receptors (*light green*) with cysteines in ECL3, 51 receptors (*cyan*) with cysteines in TM7, seven receptors (*purple*) with cysteines in both ECL1 and ECL2, two receptors (*slate gray*) with cysteines in TM5 and TM7, and one receptor (*brown*) with cysteines in both ECL2 and TM7. *B*, summary of the consistency between AlphaFold2 predictions and experimentally determined 3D structures (Cryo-EM or XFEL), focusing on the presence or absence of disulfide bonds involving N-terminal cysteines. *C*, rooted phylogenetic tree of class A GPCRs, excluding odorant receptors. Receptors with N-terminal cysteines are indicated by colored labels, with each color corresponding to the disulfide bond locations described in *panel A*. Asterisks denote IPAR1, PD2R2, and AGTR1, which are discussed in the main text. GPCR, G protein-coupled receptor; XFEL, X-ray free-electron laser.

Among the 289 human class A non-OR GPCRs cataloged in GPCRdb (18, 19), 146 (50.5%) contain at least one cysteine in the N-terminal region preceding the first transmembrane helix (TM1) (Fig. 1*A*). Structural data deposited in the Protein Data Bank (PDB) (as of January 29, 2025) showed that 29 of these GPCRs were experimentally shown to form disulfide bonds involving an N-terminal cysteine (Fig. 1*B*).

To systematically evaluate potential disulfide pairing, we analyzed the AlphaFold2 structural models of the 146 GPCRs with N-terminal cysteines. In 86 cases (58.9%), an N-terminal cysteine was predicted to form a disulfide bond with another cysteine in the extracellular region (Fig. 1*A* and Table S1). Notably, three GPCRs (MC4R, MRGX2, and GPR34) displayed N-terminal disulfide bonds in the experimental structures, although these were not predicted in the AlphaFold2 models (Fig. 1*B*). This discrepancy may reflect the conformational changes induced by ligand binding.

### Comparison with canonical bonds and structural validation

Most of the 86 GPCRs (73/86) retained the typical disulfide bond between Cys^3.25^ and Cys^45.50^, indicating that the N-terminal cysteine provides an additional extracellular linkage. In contrast, 13 GPCRs (*e*.*g*., MSHR and S1PR4) lack one or both of these conserved cysteines; in these cases, the N-terminal cysteine is predicted to form an alternative disulfide bond with other extracellular domains, potentially forming a cap over the ligand-binding pocket that contributes to receptor stability.

To further assess the accuracy of the Alphafold2 predictions, we compared its models with GPCR structures determined after the AlphaFold2 training cutoff. Among the 146 GPCRs with N-terminal cysteines, 39 had relevant structural data in the PDB (https://www.rcsb.org/, last accessed on February 28, 2024). In 30 of these cases, the predicted disulfide pattern agreed with the experimental structure (Fig. 1*B* and Table S1). For example, TAAR family proteins generally contain two N-terminal cysteines: one forms a disulfide bond with ECL1 and the other with ECL2 (highlighted in purple in Fig. 1*A*) (17). Similarly, structural studies of LPAR1 (PDB: 4Z34), PD2R2 (PDB: 7M8W), and AGTR1 (PDB:7F6G) have demonstrated N-terminal disulfide bonds with cysteines in ECL2, TM5, and TM7, respectively, supporting the structural relevance of the predicted interaction (marked with an asterisk in Figs. 1*C* and S1, C and E) (20, 21, 22).

### Evolutionary distribution of N-terminal disulfide bonds

We examined whether N-terminal disulfide bonds correlate with the evolutionary relationships among GPCRs. Phylogenetic analysis revealed that such bonds are conserved in specific families (*e*.*g*., TAARs and CCRs) but are sporadically distributed among many others (Fig. 1*C*). These findings suggest that disulfide bonds involving N-terminal cysteines arose independently multiple times during GPCR evolution, although some lineages appear to have preserved them owing to functional constraints.

### Analysis of N-terminal disulfide bonds in class B GPCRs

As an additional analysis, we examined class B GPCRs using the AlphaFold structural models. Several receptors were predicted to form disulfide bonds involving N-terminal cysteines (*e*.*g*., with TM1 or TM4 residues); however, these predictions could not be confidently evaluated because some models included signal peptide regions, and the corresponding cysteine residues were unresolved in the available experimental structures (Table S2). Notably, we did not identify any predicted intramolecular disulfide bonds involving ECL2 in the class B GPCRs. This contrasts with class A GPCRs, in which ECL2-associated disulfide bonds are commonly observed, suggesting that such disulfide bonding patterns may be characteristic structural features of class A GPCRs.

### Predicted N-terminal disulfide bonds in ORs

**Figure 2:**
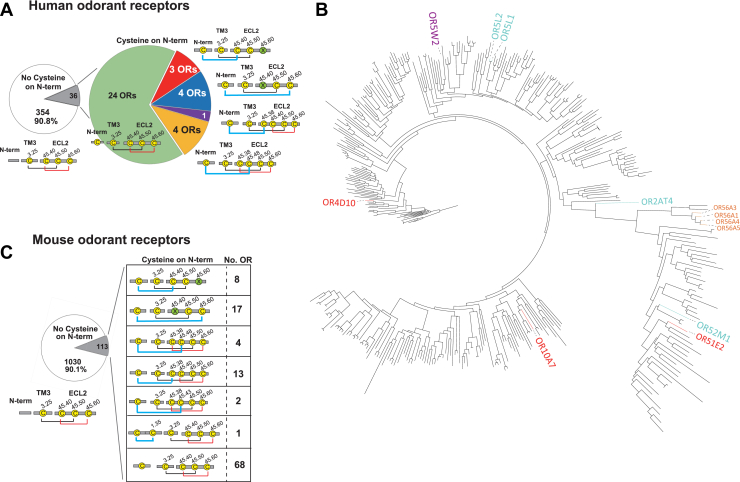
**Human and mouse odorant receptors (ORs) with cysteine residues in the N-terminal region.***A*, among the 390 human ORs, 36 receptors possess a cysteine residue in the N-terminal region. Of these, 4 ORs (*blue*) lack the cysteine closest to transmembrane domain 3 (TM3) among the three conserved cysteines on extracellular loop 2 (ECL2), while the other three ORs (*red*) lack the cysteine farthest from transmembrane domain 3 (TM3). In addition, five ORs contained an extra cysteine residue in ECL2. One of the ORs has cysteine at BW45.38, while the other four ORs have that residue at BW45.48. The remaining 24 ORs (*green*) retained all three conserved cysteines, which are typical of most ORs. In terms of disulfide bonding, the N-terminal cysteine in the *blue group* forms a bond with the third cysteine on ECL2, the *red group* with the first cysteine on ECL2, and the *orange group* with the additional cysteine described previously. In contrast, the N-terminal cysteine in the *green group* did not form any disulfide bonds. *B*, rooted phylogenetic tree of human ORs. Colored labels indicate the position of each receptor corresponding to the groups described in Figure. 2*A*. *C*, among 1143 mouse ORs, 113 receptors possess a cysteine residue in their N-terminal region. Among these, 17 ORs lacked the cysteine closest to TM3 among the three conserved cysteines on ECL2, and eight ORs lacked the cysteine farthest from TM3. Another 19 ORs had one additional cysteine on ECL2, whereas one OR had an extra cysteine on TM1. The remaining 68 ORs contained the three conserved cysteines on ECL2, consistent with the typical OR structure. All groups, except for the 68 ORs with three conserved cysteines on ECL2, formed an intramolecular disulfide bond between the N-terminal cysteine and another cysteine.

Next, we examined the presence of disulfide bonds involving N-terminal cysteines in ORs using Alphafold2. OR51E2 has revealed a disulfide bond between Cys4 in the N-terminal region and Cys^45.40^. This is consistent with the published cryo-EM structure. Encouraged by this agreement, we extended our analysis to 390 human ORs listed in The Human Olfactory Data Explorer database (23). Among these, 36 ORs (9.2%) contained at least one cysteine in the N-terminal region (Fig. 2*A* and Table S3), and 12 were predicted to form intramolecular disulfide bonds linking the N-terminal region to ECL domains.

### Patterns of alternative disulfide formation

Distinct patterns were observed depending on which conserved cysteines in ECL2 were present.

• In three ORs (OR4D10, OR10A7, and OR51E2) lacking Cys^45.60^, the N-terminal cysteine formed a bond with Cys^45.40^ (red in Figs. 2, *A* and *B*, and S3*A*).
• In four ORs (OR52M1, OR5L1, OR5L2, and OR2AT4) lacking Cys^45.40^, the N-terminal cysteine bonded with Cys^45.60^ (blue in Figs. 2, *A* and *B*, and S3*B*).
• In four closely related ORs (OR56A1, OR56A3, OR56A4, and OR56A5) that contain all three conserved ECL2 cysteines, a third disulfide bond was predicted between the N-terminal cysteine and Cys^45.48^ (orange in Figs. 2, *A* and *B*, and S3*C*).
• OR5W2 also showed a similar additional linkage with Cys^45.38^.

In contrast, the remaining 24 ORs retained the typical three ECL2 cysteines without forming additional N-terminal disulfide bonds (green in Figs. 2*A* and S3*D*).

Phylogenetic analysis indicated that N-terminal disulfide bonds in ORs are generally scattered across the phylogeny, with the exception of the OR56A family, in which these bonds are conserved (Fig. 2*B*). This pattern suggests that N-terminal disulfide bonds have arisen independently multiple times during OR evolution.

To test whether such bonds are conserved across species, we examined 1143 mouse ORs listed in the Human Olfactory Data Explorer. Of these, 113 (9.9%) contained a cysteine in the N-terminal region, a proportion similar to that in humans (Fig. 2*C* and Table S3). Among the 12 human ORs with predicted N-terminal disulfide bonds, 10 have mouse orthologs or closely related receptors with equivalent disulfide linkages, indicating that these structural features are at least partially conserved in mammalian OR evolution.

Taken together, our analysis shows that ORs lacking one of the conserved cysteines in ECL2 frequently compensate for this by forming disulfide bonds with an N-terminal cysteine. Such bonds appear to have emerged repeatedly and independently, suggesting that alternative disulfide bonding involving the N-terminal domain can be advantageous in certain cases.

### Importance of N-terminal cysteine in OR51E2

**Figure 3:**
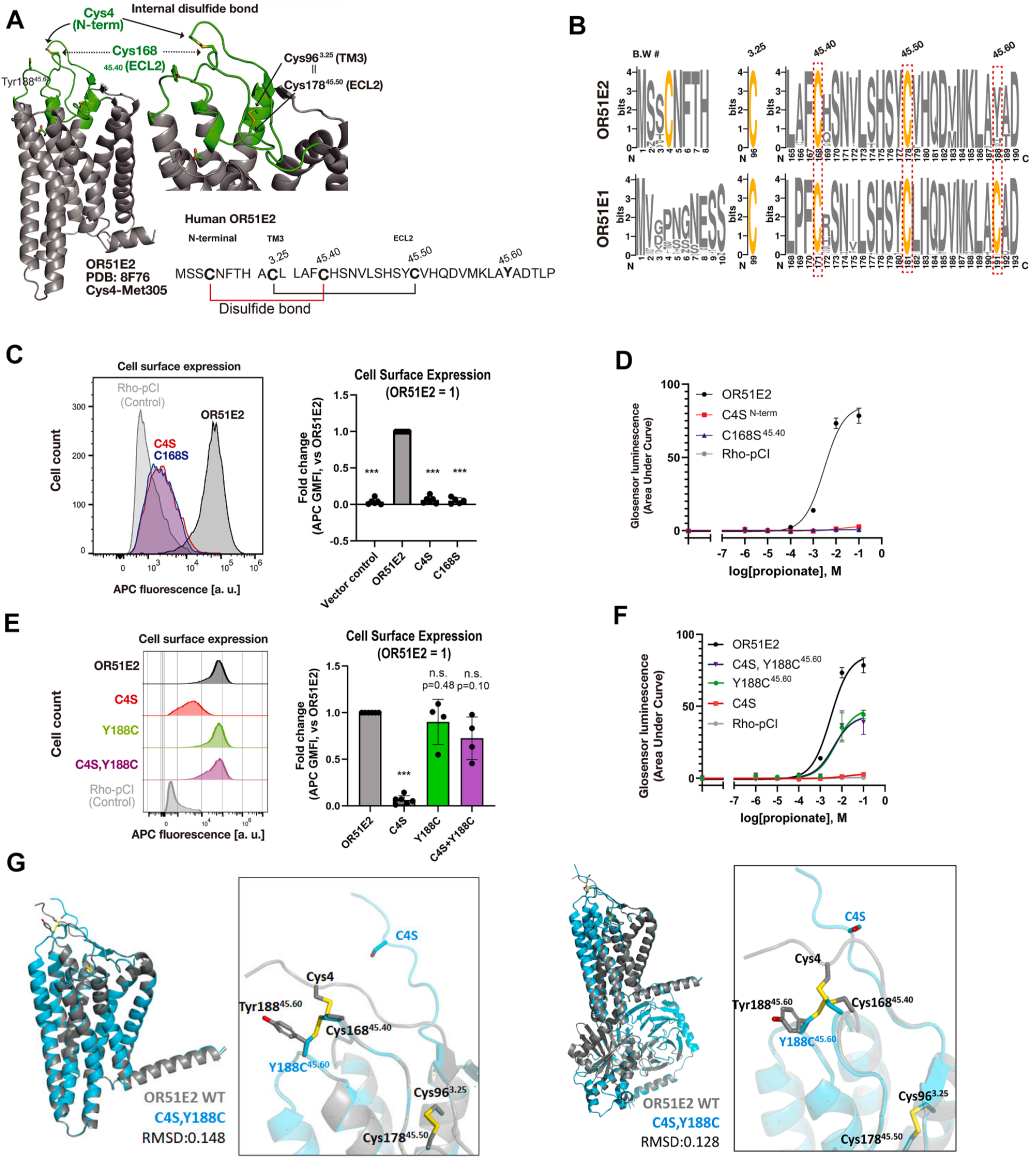
**Importance of the N-terminal cysteine in human OR51E2.***A*, structure of OR51E2 (PDB: 8F76) showing two intramolecular disulfide bonds: Cys4–Cys168 and Cys96–Cys178. The N-terminal region and ECL2 are highlighted in *green*, and disulfide bonds are highlighted in *yellow*. *B*, conservation analysis of the N-terminal, TM3, and ECL2 sequences in mammalian orthologs of OR51E2 (*top panel*) and OR51E1 (*bottom panel*). All four cysteines (*orange*) involved in the two disulfide bonds are conserved in OR51E2 orthologs, whereas the N-terminal cysteine is not conserved in OR51E1. In contrast, Cys45.60 is conserved among all OR51E1 orthologs. *C*, *left*: cell surface expression of OR51E2 WT (*black*), C4S (*red*), C168S (*blue*), and Rho-pCI vector control (*gray*) in HEK293T cells, measured by APC fluorescence. A total of 10,000 viable, single, GFP-positive cells were analyzed. *Right*: quantification of the expression reproducibility. Relative GeoMean values were normalized to 1 (OR51E2 WT). Error bars: S.D. (n = 4). paired two-tailed t-tests (∗∗∗*p* < 0.001). *D*, dose-response to propionate for OR51E2 WT (*black*), C4S (*red*), C168S (*blue*), and vector control (*gray*). Error bars: S.D. (n = 3). One-way ANOVA (*p* < 0.001), followed by Dunnett’s test (∗∗∗*p* < 0.001) for each concentration. *E*, *left*: cell surface expression of OR51E2 WT (*black*), C4S (*red*), C4S + Y188C (*purple*), Y188C (*green*), and vector control (*gray*). The same measurement conditions as in Figure 3*C*. *Right*: reproducibility data with GeoMean values normalized to 1 (OR51E2 WT). Error bars: S.D. (n = 4). One-way ANOVA (*p* < 0.001) and Dunnett’s test (∗*p* < 0.05, ∗∗∗*p* < 0.001). *F*, dose-response to propionate for OR51E2 WT (*black*), C4S (*red*), C4S + Y188C (*purple*), Y188C (*green*), and vector control (*gray*). Error bars: S.D. (n = 3). *G*, *left*: structural alignment of OR51E2 WT (*gray*) and C4S + Y188C mutant (*cyan*) models predicted by AlphaFold3. RMSD was calculated using PyMOL. *Right*: AlphaFold3-predicted activated-state models of OR51E2 WT and C4S + Y188C mutant in complex with propionate and the Golf heterotrimer (Gαolf, Gβ, and Gγ). The positions of C4, C168, and Y188 in WT (*gray*) and S4 and C188 in the mutant (*cyan*) are shown. The predicted disulfide bonds are highlighted in *yellow*. APC, allophycocyanin; ECL, extracellular loop; OR, odorant receptor; PDB, Protein Data Bank.

To investigate the role of N-terminal cysteine in ORs, we combined experimental analyses of point mutants in heterologous cells with structural predictions generated by AlphaFold2. OR51E2 is the only native OR with available Cryo-EM structural information and is among the 12 human ORs predicted to form an intramolecular disulfide bond between the N-terminal cysteine and a cysteine on ECL2 (14). Analysis of the OR51E2 structure (PDB: 8F76) aligned with the AlphaFold2 model confirmed the formation of an intramolecular disulfide bond between C4^N−term^ and C168^45.40^ (Fig. 3*A*). The amino acid at BW45.60, a typical site for a disulfide bond with the conserved cysteine on ECL2, is Y188^45.60^ in human OR51E2 (Fig. 3*A*). Alignment of mammalian OR51E2 orthologs revealed that C4 and C168 are conserved across all orthologs, whereas the amino acid at BW45.60 varied among Y, F, H, and S but was never C (Fig. 3*B*). In contrast, OR51E1 orthologs showed a more common pattern of cysteine conservation at BW45.60 and the absence of cysteine in the N-terminal region.

To experimentally evaluate the importance of the predicted disulfide bond, we generated OR51E2 mutants at C4 and C168 residues. Both mutants exhibited a significant decrease in cell surface expression and loss of agonist response in HEK293T cells (Fig. 3, *C* and *D*), demonstrating that these cysteines are essential for OR51E2 functionality. The Y188C single mutation did not affect cell surface expression but reduced the agonist response (green in Fig. 3, *E* and *F*). Substituting Y188 with A, F, H, or S did not significantly alter the agonist response, indicating that the specific amino acid at BW45.60 does not critically influence OR51E2 functionality (Fig. S4). To determine whether the expression defect caused by the C4S mutation could be rescued by introducing a cysteine at BW45.60, we generated an OR51E2 C4S + Y188C double mutant. Expression of the C4S + Y188C mutant restored cell surface localization and partially recovered the agonist response to the propionate. (purple in Fig. 3, *E* and *F*). No changes were observed in agonist selectivity (Fig. S5) or obvious agonist-induced receptor internalization was observed in either WT OR51E2 or cysteine-substituted mutants (Fig. S6). Furthermore, the AlphaFold3 models of OR51E2 and its mutants in complex with propionate and the Gαolf heterotrimer revealed highly similar overall conformations, with no obvious structural differences that could readily explain the altered signaling efficacy observed among the mutants (Fig. 3*G*). These findings suggest that the effects of N-terminal cysteine repositioning are primarily related to receptor folding and functional expression rather than ligand selectivity or receptor internalization.

Similar results were obtained with the mouse ortholog, where cell surface expression of Y188C and C4S + Y188C mutants matched WT levels, but the agonist response was only partially restored compared to WT (Fig. S7). These findings indicate that although the conserved disulfide bond among ORs is not essential for cell membrane localization, the specific bond between C4 and C168 is critical for an effective agonist response.

Consistent with these experimental results, AlphaFold3 predicted that the C4S + Y188C mutant formed a disulfide bond between C168 and C188 (Fig. 3*G*). The overall structure of this mutant closely resembled that of WT OR51E2, with an RMSD of 0.148 Å relative to the WT model, compared with 0.240 and 0.248 Å for the single mutants (C4S and C168S, respectively). Although the absolute differences in RMSD values are small and within the expected variability of computational models, and thus should be interpreted with caution, they are consistent with the idea that the C4S + Y188C mutant improves the global fold of OR51E2. AlphaFold2 models predicted differences in the pLDDT values within the extracellular region among the WT, C4S, and C4S + Y188C receptors (Fig. S8 and Table S4). Although pLDDT does not directly reflect structural stability, these observations are consistent with the altered local structural organization caused by the disruption and restoration of the predicted disulfide bond.

Similarly, using AlphaFold3, we predicted the structure of OR51E2 in the activated state by modeling its complex with propionate and mini-Gαolf (Fig. 3*G*). All five predicted models consistently showed the formation of a disulfide bond between C4 and C168, indicating that agonist binding did not induce notable structural changes (Fig. S9). Moreover, no significant difference in RMSD was observed between the WT and C4S/Y188C mutant (RMSD = 0.128 Å).

In summary, these results suggest that the C4^N−term^ in OR51E2 is a critical residue that forms an essential intramolecular disulfide bond with Cys168^45.40^ in ECL2. This bond is critical for the proper folding and functional expression of OR51E2 and contributes to robust agonist responsiveness.

### Cysteine position dependency in the N-terminal region of OR51E2

**Figure 4:**
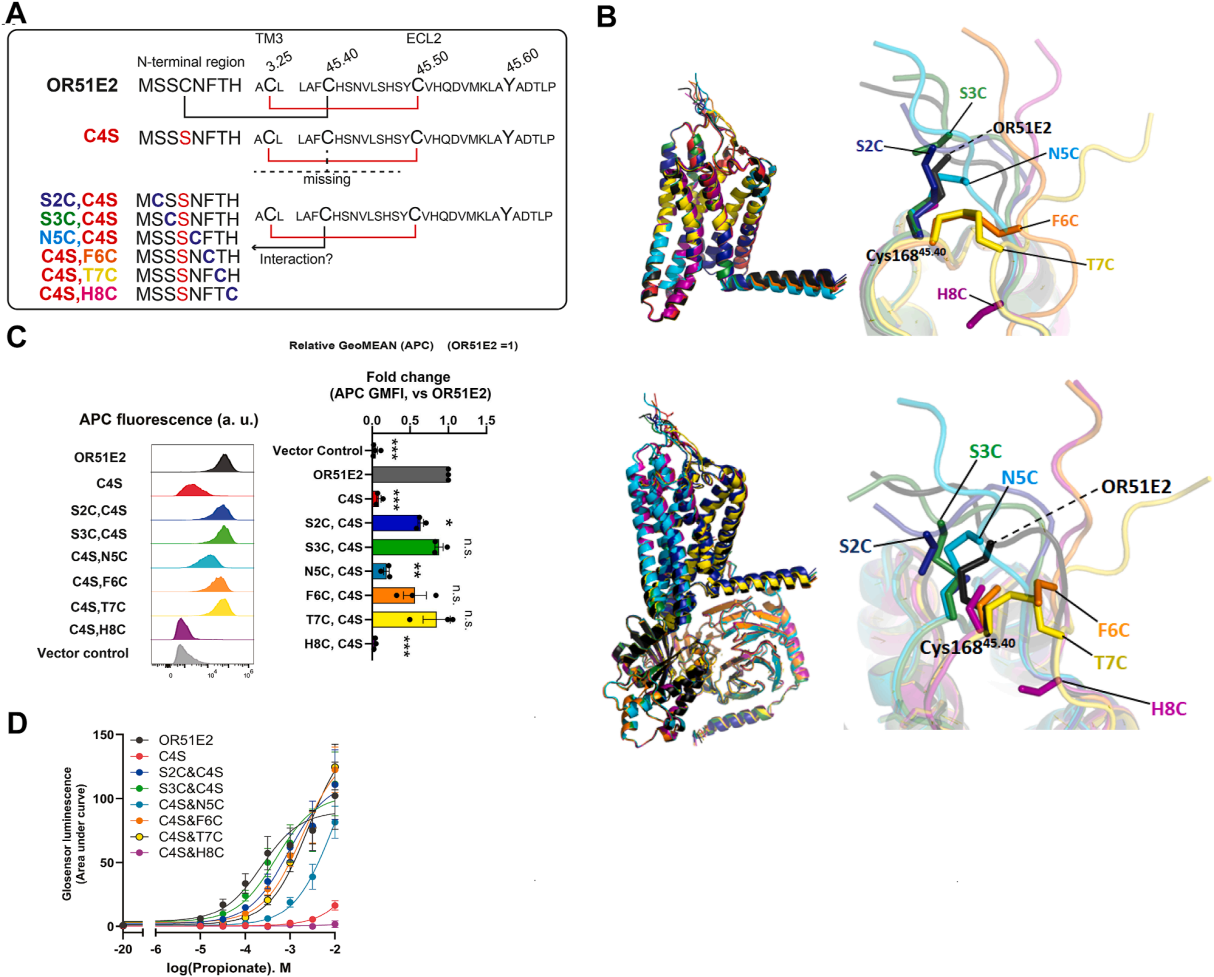
**Optimal position of N-terminal cysteine in human OR51E2.***A*, N-terminal amino acid sequences of OR51E2 mutants designed to assess the impact of cysteine positioning: C4S, S2C + C4S, S3C + C4S, C4S + N5C, C4S + F6C, C4S + T7C, and C4S + H8C. Cysteines (*blue*) and serines (*red*) were introduced at specific positions. *B*, structural comparison of OR51E2 and its mutants. *Upper*: OR51E2 WT (*black*) and each mutant (colors as in A), with predicted models generated using AlphaFold3. N-terminal disulfide formation of each mutant is shown *left*. *Lower*: N-terminal disulfide bonding formation of receptors in complex with Golf heterotrimers and propionic acid. *C*, *left*: cell surface expression of WT, mutants, and vector control in HEK293T cells, assessed by APC fluorescence. A total of 10,000 viable GFP-positive cells were analyzed. *Right*: expression reproducibility normalized to WT (1.0). Error bars: S.D. (n = 3). paired two-tailed t-tests (∗*p* < 0.05, ∗∗*p* < 0.005,∗∗∗*p* < 0.001). *D*, dose-response curves for propionate in WT, mutant, and vector control cells. APC, allophycocyanin; OR, odorant receptor.

Introducing a mutation at the C4^N−term^ in OR51E2 significantly reduced its functional expression (Fig. 3, *C* and *D*), indicating that C4 is a critical residue for OR51E2 functionality. We hypothesized that OR51E2 has evolved to position C4 optimally within the N terminus. To test whether the fourth position is indeed optimal for the N-terminal cysteine, we experimentally assessed OR51E2 mutants with cysteine substitutions at different N-terminal positions for heterologous cell surface expression in HEK293T cells (Fig. 4*A*). Cell membrane localization analysis revealed that the S2C + C4S, S3C + C4S, C4S + F6C, and C4S + T7C mutants achieved significant membrane localization, although at lower levels than the WT OR51E2 (Fig. 4*C*). In contrast, the C4S + H8C mutant did not exhibit cell membrane localization (Fig. 4*C*). When the agonist response to propionic acid was evaluated, mutants with cell membrane localization showed response levels comparable to that of the WT (Fig. 4*D*). A positive correlation was observed between the relative changes in cell membrane localization and agonist response strength (Spearman’s test, r = 0.89, *p* < 0.001) (Fig. S10*A*), indicating that the presence of cysteine at the appropriate N-terminal position is critical for the functional expression of OR51E2. Notably, the C4S + N5C mutant failed to localize to the membrane, likely due to the loss of N-glycosylation at N5 (Fig. 4*C*) (24). Comparing cell membrane localization and agonist response, WT OR51E2 with a cysteine at the fourth position demonstrated the highest signal-to-noise ratio at the lowest DNA amount used for transfection.

Structural models indicate that when a cysteine is placed at positions two to seven in the N-terminal region, a disulfide bond is formed between C^N-term^ and C168^45.40^ (Fig. 4*B*). However, in the C4S + H8C mutant, Cys8 and Cys168 were not predicted to form a disulfide bond (Fig. 4*B*, purple). Differences in the pLDDT values were also observed among the models (Fig. S11*A* and Table S4), consistent with the structural changes associated with the disruption of the predicted N-terminal–ECL2 disulfide bond. In summary, when integrated with the experimental data, these findings are consistent with the hypothesis that C4 is the optimal site for the functional expression of OR51E2. Both mutational analyses and structural modeling highlight the critical role of the intramolecular disulfide bond mediated by the N-terminal C4, a conserved feature among mammalian OR51E2, in enabling proper receptor functions.

### N-terminal cysteine compensates for the loss of a conserved intramolecular disulfide bond

**Figure 5:**
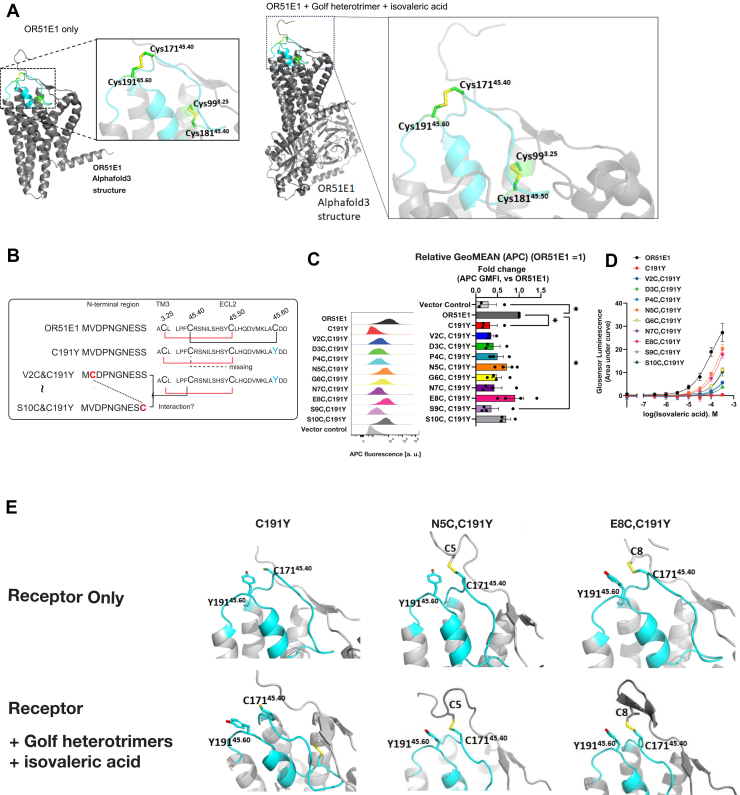
**Introduction of an N-terminal cysteine rescues the loss of ECL2 cysteine in human OR51E1.***A*, *left*: predicted structural model of OR51E1 WT generated using AlphaFold3. *Right*: predicted structural model of OR51E1 WT in complex with Golf heterotrimer and isovaleric acid yielded using Alphafold3. *B*, N-terminal amino acid sequences of OR51E1 mutants constructed to assess the effect of introducing cysteine into the N-terminal region. All mutants carried the C191Y substitution, with additional cysteine introduced at positions 2 to 10 (V2C, D3C, P4C, N5C, G6C, N7C, E8C, S9C, and S10C). Artificially inserted tyrosines (*blue*) and cysteines (*red*) are highlighted in the figure. *C*, *left*: cell surface expression of OR51E1 WT, mutants, and vector control in HEK293T cells, measured *via* APC fluorescence. A total of 10,000 viable, GFP-positive cells were analyzed. *Right*: relative GeoMean values were normalized to 1 (OR51E1 WT). Error bars represent the SD (n = 3). paired two-tailed *t* test (∗*p* < 0.05). *D*, *Left*: dose-response curves for isovaleric acid for OR51E1 WT, C191Y, and other mutants. Error bars: S.D. (n = 4). *E*, *upper*: structural models of selected mutants: C191Y (showing C171 and Y191), N5C + C191Y (C5, C171, Y191), and S8C + C191Y (C8, C171, Y191), generated using AlphaFold3. *Lower*: structural models of selected mutants in complex with Golf heterotrimers and 2-methyl-2-thiazoline generated using AlphaFold3. All models show only the *upper areas* of the receptors. APC, allophycocyanin; ECL, extracellular loop; OR, odorant receptor.

The critical role of the N-terminal cysteine in OR51E2 raises the possibility that ORs can compensate for the absence of the conserved disulfide bond between Cys^45.40^ and Cys^45.60^ if an appropriately positioned N-terminal cysteine interacts with ECL2 of the OR. To test this hypothesis, we investigated whether introducing a cysteine into the N-terminal region could restore the functional expression of ORs, where the loss of the conserved disulfide bond led to reduced functionality. For this study, OR51E1 was selected as a model because of its similarity to OR51E2 and the presence of two intramolecular disulfide bonds formed by C172^45.40^ and C191^45.60^ in ECL2, which are characteristic of typical ORs (Fig. 5, *A* and *B*).

Mutating C191^45.60^ in OR51E1 to tyrosine (C191Y) significantly decreased cell membrane localization, supporting the essential role of C191 in OR51E1 structural integrity (Fig. 5*C*, red). To evaluate whether the lost functional expression of the C191Y mutant could be restored by introducing a cysteine in the N-terminal domain, we introduced cysteine residues at various positions, constructing ten mutants by substituting each of the first 11 N-terminal amino acids of OR51E1 C191Y^45.60^ with cysteine (Fig. 5*B*). We then evaluated the cell surface expression of each mutant. Both N5C + C191Y and E8C + C191Y mutants restored functional expression (Fig. 5, *C* and *D*). Other mutants also demonstrated partial recovery in cell surface expression, with a strong positive correlation between membrane localization and agonist response (Spearman’s test, r = 0.92, *p* < 0.001) (Fig. S10*B*). Therefore, these experiments provide insights into how N-terminal-ECL2 disulfide connectivity can modulate both receptor stability and functional conformation.

To further assess the effect of introducing N-terminal cysteines at various positions of OR51E1 *in silico*, we examined whether AlphaFold3 predicted that each introduced N-terminal cysteine would form a disulfide bond with C172^45.40^ in the OR51E1 C191Y^45.60^ mutant (Fig. 5*E*). Differences in the pLDDT values were observed among the AlphaFold2 models (Fig. S11*B* and Table S4). However, despite relatively high confidence scores, the N7C + C191Y and S9C + C191Y mutants showed limited recovery of cell surface expression, suggesting a potential contribution of N-glycosylation to OR51E1 membrane trafficking.

In summary, introducing a cysteine into the N-terminal region can stabilize the structure between the N terminus and ECL2, compensating for the absence of intramolecular disulfide bonds within ECL2 and restoring functional expression. These findings highlight the potential of leveraging N-terminal modifications to partially restore ORs function.

### The importance of third intramolecular disulfide bond in the mouse OR

**Figure 6:**
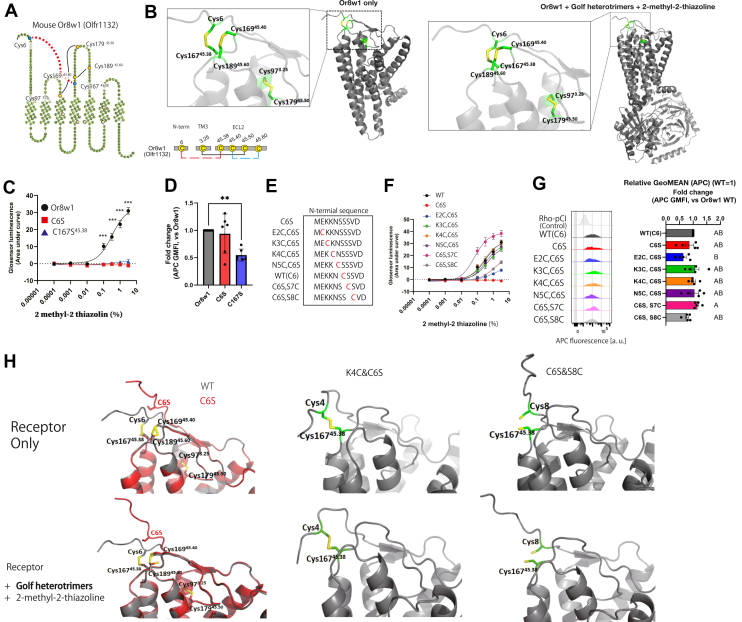
**ORs forming a third intramolecular disulfide bond *via* N-terminal cysteine.***A*, snake plot of Or8w1 showing the amino acid sequence and position of cysteines. *Blue circles* indicate cysteines involved in a disulfide bond between the N terminus and ECL2, and *yellow circles* indicate other cysteines. *B*, *upper right*: predicted structure of Or8w1 generated using AlphaFold3, showing the positions of C6, C97, C167, C169, C179, and C187 and three disulfide bonds (*yellow*). *Left*: predicted structure of the complex of Or8w1, Golf heterotrimer, and 2-methyl-2-thiazoline, showing the positions of C6, C97, C167, C169, C179, and C187 and three disulfide bonds (*yellow*). *Lower right*: schema illustrating the specific disulfide bond pairings among the labeled cysteines. *C*, dose-response analysis of Or8w1 WT (*black*), C6S (*red*), and C167S (*blue*) in response to 2-methyl-2-thiazolin. Error bars: S.D. (n = 3). Statistical significance of differences from the WT was evaluated using One-way ANOVA (*p* < 0.001), followed by Dunnett’s test (∗∗∗*p* < 0.001). *D*, cell surface expression of WT, C6S, and C167S mutants. GeoMean values were normalized to 1 (WT). Error bars: S.D. (n = 3). One-way ANOVA (*p* < 0.001) followed by Dunnett’s test (∗∗∗*p* < 0.001). *E*, N-terminal amino acid sequences of Or8w1 mutants designed to examine the effect of cysteine positioning. *F*, *left*: dose-response curves of Or8w1 mutants exposed to 2-methyl-2-thiazolin. *G*, *left*: cell surface expression of Or8w1 mutants, including double mutants from Figure 6*E* and vector control (*gray*), measured in HEK293T cells *via* APC fluorescence. *Right*: relative GeoMean values normalized to WT (1). Error bars represent the SD (n = 5). One-way ANOVA (*p* < 0.001) followed by Tukey’s test. *H*, structural comparison of Or8w1 and its mutants. *Left*: overlay of the WT (*gray*) and C6S (*red*) models. *Center*: positions of C4 and C167 in the K4C + C6S double mutant; *Right*: C8 and C167 in the C6S + S8C mutant. The structures of the mutants were generated using AlphaFold 3. The *upper figures* show the N-terminal disulfide bond formation of receptors without additional molecules. The *lower figures* show the N-terminal disulfide bonding formation of receptors in complex with Golf heterotrimers and 2-methyl-2-thiazoline. APC, allophycocyanin; OR, odorant receptor.

To further explore the role of disulfide bonds between the N-terminal region and ECL2, we focused on mouse Or8w1 (Olfr1132), an OR that responds to the agonist, 2-methyl-2-thiazoline (2-MT). AlphaFold3 predictions revealed that Or8w1 forms an unconventional intramolecular disulfide bond between C6^N−term^ and C167^45.38^ in ECL2 (Fig. 6, *A* and *B*).

To evaluate the functional importance of this disulfide bond, we constructed C6S and C167S mutants. Both mutants exhibited a significant reduction in their response to 2-MT (Fig. 6*C*), indicating that the disulfide bond predicted by AlphaFold3 is critical for Or8w1’s functional expression in heterologous cells. Notably, the cell surface expression level of the C6S mutant was comparable to that of the WT receptor, whereas the C167S mutant showed a marked reduction (Fig. 6*D*), suggesting that disruption of the predicted third disulfide bond impairs functional receptor expression through effects on receptor conformation, responsiveness, and/or trafficking.

Similar to our analysis of OR51E2 (Fig. 4), we examined the positional requirements of the N-terminal cysteine by introducing cysteine residues at alternative positions within the N-terminal region (Fig. 6*E*). The mutants K3C (green), K4C (orange), N5C (purple), and S7C (pink) rescued the agonist response lost due to the C6S^N−term^ mutation (red), with the C6S + S7C mutant showing a higher response than the WT (pink in Fig. 6*F*). In contrast, the E2C and S8C mutants failed to rescue agonist responsiveness. Although the E2C + C6S mutants showed little reduction in cell surface expression (Fig. 6*G*), they responded only to higher concentrations of 2-MT, indicating partial functional recovery. Importantly, other mutations did not cause substantial changes in cell surface expression compared to the WT receptor, indicating that membrane localization was largely preserved (Fig. 6*G*). Instead, the primary effect was on agonist responsiveness, suggesting that repositioning of the N-terminal cysteine can restore receptor function without significantly affecting trafficking.

Structural models generated using AlphaFold3 revealed that the loss of the disulfide bond in the C6S mutant increased the distance between the N-terminal region and ECL2 (red in Fig. 6*H*). In contrast, the K4C + C6S mutant, which restored functional expression, formed an intramolecular disulfide bond between C4 and C167 (Fig. 6*H*). The C6S + H8C mutant, which exhibited poor functional expression, failed to form a disulfide bond between C8 and C167 (Fig. 6*H*). AlphaFold2 models predicted relatively higher N-terminal pLDDT values for variants capable of forming N-terminal–ECL2 disulfide bonds, whereas lower values were observed for mutants with impaired function (Fig. S11*C* and Table S4). These observations are consistent with the structural role of the N-terminal–ECL2 disulfide bond in Or8w1.

Together with the successful rescue observed upon reintroduction of cysteine residues at nearby N-terminal positions, these findings suggest that the presence of an N-terminal cysteine capable of forming a disulfide bond with C167^45.38^ is important for the efficient functional expression of Or8w1.

Collectively, these findings suggest that some ORs possess N-terminal cysteines capable of forming diverse intramolecular disulfide bonds with the ECL2. While ORs have evolved rapidly to respond to a wide array of odorants, they have also evolved flexible, variable, and disulfide-bonding architectures that support receptor functional expression and odorant responsiveness.

## Discussion

Proteins must fold correctly to ensure their proper functioning. One mechanism that helps maintain structural integrity and ensures biological activity is the formation of intramolecular disulfide bonds. GPCRs utilize this mechanism through disulfide bonds between conserved cysteines located in their extracellular domains (11). Mammalian ORs, which comprise approximately half of the human GPCR repertoire with approximately 400 members (1, 2, 25), share conserved residues, including several cysteines essential for their overall structure and function (12). Although the necessity of correct folding for the functional expression of ORs is well acknowledged, the specific structural features critical for this process remain unclear. In addition, while conserved residues support common structural frameworks, sequence variations at less-conserved positions often underlie functional diversity (26), and even single amino acid changes can dramatically alter receptor function (27, 28). In this study, we explored the roles of atypical cysteine residues in the N-terminal extracellular domain of GPCRs, focusing on a set of ORs, and identified a strategy in the largest GPCR family that enables alternative disulfide bond formation within individual ORs.

One important contributor to the correct folding of GPCRs is the formation of intramolecular disulfide bonds. In class A GPCRs, a conserved disulfide bond between Cys^3.25^ and Cys^45.50^ stabilizes the ECL 2^11^. However, the role of cysteines in the N-terminal extracellular domain remains understudied. Historically, structural studies have often omitted this and other flexible regions to facilitate crystallization. Advances in cryo-EM have enabled the visualization of intact GPCR structures, revealing disulfide bonds involving the N-terminal domain. For instance, the recent structural elucidation of TAAR9 showed an intramolecular disulfide bond formation between the N-terminal domain and an ECL, with mutation of the N-terminal cysteine significantly diminishing receptor function (17).

In this study, our protein sequence-based analysis revealed that a minor yet significant fraction of GPCRs, including ORs, possess one or more cysteines in their N-terminal domain. Our meta-analysis of experimentally determined structures, combined with computational structural predictions using AlphaFold, suggests that many of these N-terminal cysteines likely form disulfide bonds with cysteines in the extracellular regions, revealing an underappreciated structural role for N-terminal cysteines in GPCRs.

We focused on OR51E2, a mammalian OR whose structure was solved using cryo-EM (14). OR51E2, which is expressed not only in olfactory neurons but also in several nonolfactory tissues, such as kidney, gut, and prostate cancer cells (6, 7, 8), exhibits higher structural stability than many other ORs, leading to the successful elucidation of its cryo-EM structure (14). Our analysis revealed the critical importance of the N-terminal cysteine in OR51E2, as mutation of this residue abolished the receptor function. This finding is consistent with prior observations in the non-OR olfactory GPCR TAAR9 (17).

Furthermore, our experimental scanning of cysteine positions within the N-terminal domain of OR51E2 revealed limited flexibility in cysteine position. Structural predictions and mutational analyses were consistent with the idea that the N-terminal-ECL2 disulfide bond contributes to functional receptor expression. However, the precise structural mechanisms underlying these effects remain unclear. In particular, several limitations should be considered when interpreting the structural and functional roles of the proposed N-terminal disulfide bonds. Structural models of the OR51E2 mutants generated using AlphaFold2 and AlphaFold3 included candidate structures both with and without the proposed disulfide bonds, even for the same receptor sequence (Fig. S9). This variation could reflect either genuine structural heterogeneity in the extracellular regions or uncertainty in the prediction of flexible extracellular structures and disulfide connectivity. Thus, current structural models do not establish whether disulfide-bonded and nonbonded receptor populations coexist in cells or whether the alternative models correspond to biologically relevant conformational states.

Importantly, we showed that the absence of the conserved cysteine in the ECL2 domain is typically accompanied by the presence of an N-terminal cysteine in OR51E2 and other ORs, suggesting that the N-terminal cysteine may serve an equivalent functional role. Supporting this, the introduction of a conserved ECL2 cysteine into OR51E2 rescued the cell surface expression and odor-induced responses of the N-terminal-cysteine mutant. These results suggest that while intramolecular disulfide bonds are essential for OR folding and function, their location can be flexible to some extent, offering an alternative structural strategy for maintaining receptor structural integrity while allowing for evolutionary adaptation.

In addition, in Or8w1, structural predictions and functional assays indicated the presence of a third intramolecular disulfide bond involving the N-terminal domain. Although the direct demonstration of this bond under physiological conditions remains to be explored, the consistent loss of function upon mutation of either cysteine suggests that such bonds may contribute to structural integrity.

Our study also revealed that intramolecular disulfide bonds involving the N-terminal domain underwent lineage-specific diversification in both ORs and non-OR class A GPCRs, potentially through repeated gain, loss, or repositioning events during evolution. While some GPCRs, such as TAARs, exhibit conserved disulfide bonds involving N-terminal cysteines among family members (17), the majority of cases display multiple, isolated emergences of such bonds. For example, the paralogous pair OR51E2, which has an N-terminal cysteine, and OR51E1, which does not, illustrate this evolutionary divergence. Interestingly, these ORs show conserved features across their orthologs, hinting at the functional importance of each receptor.

Although engineering OR51E2 to form a disulfide bond, consistent with those of other ORs rescued the loss of function caused by removal of the N-terminal cysteine, it did not fully restore the native receptor function, suggesting that this atypical disulfide bond is specifically required for full activity. Similarly, introducing an N-terminal cysteine partially, but not fully, rescued the loss of function caused by the mutation of the conserved ECL2 cysteine in OR51E1. We propose that the use of alternative disulfide bonds represents a strategy by which ORs diversify their functions, increase their tolerance to mutations, and promote rapid functional evolution. The observation that some cysteine mutants showed altered ligand-induced responses despite having comparable cell-surface expression levels further suggests that extracellular cysteine residues and their associated disulfide-bonding arrangements may have functional roles beyond supporting receptor folding and trafficking (Figs. 3, *E* and *F*, 6, *C* and *D*). They may affect the conformation of the extracellular receptor region (13), stabilize an activation-competent receptor state, or contribute to conformational coupling between the extracellular region and transmembrane core (14). Nevertheless, because the present assays measure downstream cAMP accumulation, they cannot distinguish whether the observed functional differences result from altered ligand binding, receptor activation, G protein coupling, or downstream signal amplification.

Building on these findings, several important avenues should be pursued in future studies. First, direct biochemical evidence of disulfide bond formation between the N-terminal cysteine and ECL cysteines in ORs will be required to validate the proposed bonding arrangements and determine their relative abundance and potential dynamics. However, such analyses remain technically challenging for ORs because of their low expression levels, hydrophobicity, and the difficulty of obtaining sufficient amounts of properly folded and functionally active receptor proteins while preserving native disulfide-bonding states. The timing, cellular location, and stability of these bonds also remain unresolved. Although extracellular disulfide bonds are generally formed during oxidative protein folding in the endoplasmic reticulum (29, 30), the present results do not determine when the proposed bonds are established during receptor biosynthesis or whether their bonding states are maintained or remodeled during receptor trafficking, membrane residence, internalization, and turnover. It also remains unknown whether the bonds themselves or their surrounding local conformations change during ligand binding and receptor activation.

Cell-based assays provide ensemble-averaged measurements of heterogeneous receptor populations that may differ in maturation, localization, disulfide-bonding state, and activation state. Conversely, structures obtained from purified-receptor using cryo-EM represent selected receptor populations stabilized under specific buffer, detergent, ligand, and protein complex conditions, which may not fully reproduce the dynamic membrane environment of living cells (14, 16, 17). Further biochemical and biophysical studies, including direct disulfide mapping (31) and time- and compartment-resolved analyses (16), will therefore be required to define the formation, maintenance, structural heterogeneity, and potential activation-dependent dynamics of these extracellular disulfide bonds. Investigating the redox sensitivity of alternative disulfide bonds may also reveal regulatory mechanisms that influence receptor function *in vivo* (32). Expanding cysteine-engineering approaches to a broader array of ORs should broaden our understanding of how alternative disulfide bonds contribute to OR stability and function, which may in turn enable stabilization of poorly expressed receptors, facilitate their functional characterization, and provide a path toward systematic deorphanization.

In conclusion, our study identified an alternative intramolecular disulfide bonding strategy involving the N-terminal domain that contributes to the functional expression and diversification of ORs and potentially other GPCRs. These findings suggest that variation in extracellular disulfide bonding arrangements provides structural and functional flexibility during OR evolution. Our findings offer new approaches for studying and engineering functional GPCRs and may help overcome major hurdles in OR expression and characterization.

### Experimental procedures

#### GPCR sequence analysis

To construct the phylogenetic tree shown in Figure 1*C*, we obtained 289 non-OR GPCR sequences from the GPCR Database (18). Multiple sequence alignment was performed using MAFFT (E-INS-I default parameters) (33) with five nonclass A GPCRs as the reference sequences. Phylogenetic analysis was conducted using IQ-TREE (version 2.3.6; (34). Bootstrap support values were calculated based on 1000 ultrafast bootstrap approximations, and branch support was further assessed using the Shimodaira-Hasegawa-like approximate likelihood ratio test. The analysis was performed using 22 threads. The resulting phylogenetic tree was visualized using iTOL v7 (35).

OR51E2 and OR51E1 ortholog proteins were searched using BLAST, and 90 and 67 orthologous amino acid sequences were obtained from NCBI, respectively. Multiple sequence alignments were performed using ClustalW. Weblogo (36) was used to visualize the conservation of amino acids. Predicted protein structures were obtained from the AlphaFold Protein Structure Database based on the corresponding amino acid sequences (37, 38).

### DNA and vector preparation

The open reading frames of the OR51E2, OR51E1, and Or8w1 genes were cloned from the human or mouse genome and subcloned into pCI (Promega) with a rhodopsin tag (Rho-tag) at the N terminus, which has been previously demonstrated to enhance the plasma membrane localization of multiple ORs (39). To generate OR mutants, DNA fragments of OR genes were amplified using appropriate primer pairs and PrimeStar MAX polymerase (Takara Bio). The fragments were mixed and amplified by PCR to obtain the full sequences. All plasmid DNA was purified using NucleoSpin plasmid transfection grade (MACHEREY-NAGEL GmbH & Co). The other expression vectors were the same as those used in a previous study (4). All plasmid sequences were verified by Sanger sequencing.

### Cell culture

HEK293T cells and Hana 3A (40) cells were grown in minimal essential medium containing 10% FBS (vol/vol) with penicillin‒streptomycin and amphotericin B. All cell lines were incubated at 37 °C, with saturated humidity and 5% CO2. No *mycoplasma* infection was detected in the cell cultures.

### Flow cytometry analysis

HEK293T cells were grown to confluence, resuspended, and seeded onto 35 mm plates at 25% confluence. The cells were cultured overnight. Rho-tagged OR in the pCI and AcGFP-N1 expression vectors (Takara Bio) were transfected using Lipofectamine 2000 transfection reagent (Thermo Fisher Scientific). After 22 to 24 h, the cells were resuspended in a cell stripper (Corning) and kept on ice. The cells were spun down at 4 °C and resuspended in PBS containing 15 mM NaN3 and 2% FBS to wash away the cell strips. They were then incubated with mouse anti-Rho4D2 primary antibody (Merck-Millipore) (41), washed, and stained with APC-conjugated goat anti-mouse IgG antibody (BioLegend) in the dark. 7-amino-actinomycin D (Merck-Millipore) was added to stain the dead cells. The cells were analyzed using a CytoFLEX flow cytometer (Beckman Coulter) with gating to allow for GFP-positive, single, spherical, and viable cells. The APC fluorescence intensities were analyzed and visualized using FlowJo software (Becton, Dickinson and Company). The FACS Analysis experiments were repeated on at least three different days. For receptor internalization analyses, transfected cells were stimulated with the indicated agonist prior to harvesting and subsequently processed for flow cytometric analysis using the staining and gating procedures described previously.

### Glosensor analysis

Propionic acid and isovaleric acid were purchased from FUJIFILM Wako Chemicals, and 2-MT was purchased from TCI Chemicals. A Glosensor cAMP Assay (Promega) was used to measure the changes in cAMP levels caused by receptor activation upon ligand binding (4). Hana3A cells were plated in poly D-lysine-coated 96-well plates. After 18 to 24 h of plating, the cells were transfected with 80 ng/well of plasmids encoding ORs, 5 ng/well of RTP1S, and 10 ng/well of Glosensor-22F plasmid (Promega). After 18 to 24 h, the medium was replaced with 25 μl of HBSS (Gibco) containing 10 mM Hepes and 1 mM glucose, followed by 25 μl of HBSS containing GloSensor cAMP Reagent (Promega). The plates were kept in the dark at RT for two hours to equilibrate the cells with the reagent. The test plate was then inserted into the plate reader. Luminescence derived from the basal activity of each OR was measured. For liquid stimulation, cells were stimulated by adding an odorant diluted in HBSS containing 10 mM Hepes and 1 mM glucose. Soon after the odorant stimulation, the test plate was inserted into a GloMax Discover Microplate Reader (Promega). The luminescence in each well was measured at 60 s intervals for 20 cycles. All luminescence values were divided by the values obtained from cells transfected with the empty vector at the same cycle. Each comparison was performed in triplicates. The Glosensor Analysis experiments were conducted in triplicate and repeated on at least three different days.

### Structure model analysis

The 3D structure data of OR51E2 (PDB: 8F76) were downloaded from PDB, OR51E1 and Or8w1 were downloaded from the AlphaFold2 database (https://alphafold.ebi.ac.uk/, downloaded 07/17/2022) (42). The structure models of each mutant were created by using the ColabFold v1.5.5: AlphaFold2 using MMseqs2.

(https://colab.research.google.com/github/sokrypton/ColabFold/blob/main/AlphaFold2.ipynb) (43). Visualization was performed using the PyMOL software.

AlphaFold2 also provides residue-level confidence scores, referred to as predicted local distance difference test (pLDDT) values. These scores reflect the algorithm’s confidence in the local structure; low scores may indicate either intrinsic flexibility/disorder or insufficient information for confident prediction, whereas high scores reflect greater prediction reliability. Importantly, the pLDDT values do not directly measure physical stability or rigidity. In this study, we used pLDDT values only in a comparative manner to evaluate differences in model confidence among variants, and any implications for structural stability were considered indirect and hypothesis-generating.

Structural models were generated using AlphaFold3 installed locally, following the publicly available instructions provided by DeepMind (9). The amino acid sequences of OR51E2 and its mutants were used as inputs. For activated-state predictions, propionate was included as a ligand using its SMILES representation along with the G-protein heterotrimer (Gαolf, Gβ1, and Gγ13). Five models were generated for each prediction condition in this study. Predicted disulfide bond formation was assessed by visually inspecting the resulting models and the spatial arrangement of the cysteine residues.

### Statistical analysis

Multiple comparisons were performed using ANOVA, followed by Dunnett’s or Tukey’s *post hoc* tests, as appropriate. For comparisons between mutant receptors and the corresponding WT receptors measured within the same independent experiment, paired two-tailed t-tests were used. Statistical analyses were performed using GraphPad Prism 10 software (GraphPad Software).

## Data availability

All relevant data are available within the article and its supporting information or from the authors upon reasonable requests.

## Supporting information

This article contains supporting information Figures S1–S5.

## Conflicts of interest

H. M. has received royalties from ChemCom, research grants from Givaudan, and consultancy fees from Kao Corporation. The remaining authors declare no conflicts of interest.
