# Supplementary material for: Alternative extracellular disulfide bond formation by N-terminal cysteines supports functional expression of odorant receptors
Source: J Biol Chem. 2026 Jul 13;302(9):113319. doi: 10.1016/j.jbc.2026.113319 (PMC13486327; doi:10.1016/j.jbc.2026.113319)
Supplement: Supplementary file 1 [file mmc1.pdf]

Supplementary information

Title

Alternative Extracellular Disulfide Bond Formation by N-terminal Cysteines Supports Functional Expression of Odorant Receptors

Authors

Nonoko Muto[1], Tomoyo Koshizawa[1], Miki Takeda[1], Xiaoyang Serene Hu[2], Ryosuke Inoue[1], Masafumi Yohda[1], Hiroaki Matsunami[2] and Yosuke Fukutani[1]

Affiliations

[1]Department of Biotechnology and Life Science, Tokyo University of Agriculture and Technology, Koganei, Tokyo 184-8588, Japan

[2]Department of Molecular Genetics and Microbiology, Duke University Medical Center,
Durham, NC 27710, USA

Contents

Figure S1 Comparison of the model structure constructed by Alphafold2 of the Human Class A GPCR with the three-dimensional structure of CryoEM, focusing on the intramolecular disulfide bond of the N-terminal cysteine.
Figure S2 The model structure constructed by Alphafold2 of the Human Class B GPCR focusing on the intramolecular disulfide bond of the cysteine on N-terminal domain.
Figure S3 Predicted structures of Human ORs which have an intramolecular disulfide bond using Alphafold2
Figure S4 Effect of Tyr188$^{45.60}$ substitution in human OR51E2
Figure S5 Ligand selectivity of wild-type OR51E2 and disulfide-bond mutants toward short-chain carboxylic acids.
Figure S6 Analysis of internalization of OR51E2 wild-type and mutants.
Figure S7 Importance of the disulfide bond between Cys4N-term and Cys16845.40 in Mouse Or51e2
Figure S8 Predicted Local Distance Difference Test (PLDDT) scores of each residue in OR51E2 WT (black), C4S (red), and C4S+Y188C (purple)
Figure S9 Structural alignment of 5 structures generated by AlphaFold3 as for human OR51E2 wild-type and mutants.
Figure S10 Correlation analysis between cell surface expression and agonist response.
Figure S11 Predicted structural stability (pLDDT scores) of each mutant

Tables S1–S5 have been uploaded as separate supplementary files. The corresponding table legends are provided below.

Table S1 Sequence alignment and predicted N-terminal disulfide-bonding patterns of human Class A GPCRs.
Sequence alignment of human Class A GPCRs obtained from the GPCRdb database. The table includes receptor names, UniProt identifiers, positions of N-terminal and extracellular cysteine residues, and predicted intramolecular disulfide-bonding partners based on AlphaFold structural models and experimentally determined structures where available.

Table S2 Sequence alignment and predicted N-terminal disulfide-bonding patterns of human Class B GPCRs.
Sequence alignment of human Class B GPCRs obtained from the GPCRdb database. The table includes receptor names, UniProt identifiers, positions of N-terminal and extracellular cysteine residues, and predicted intramolecular disulfide-bonding partners based on AlphaFold structural models and experimentally determined structures where available.

Table S3 Sequence alignment and cysteine distribution of human and mouse odorant receptors
Sequence alignments of human and mouse odorant receptors were obtained from The Human Olfactory Data Explorer (HORDE) database. The table includes receptor names, HORDE identifiers, positions of cysteine residues in the N-terminal region and extracellular domains, and the presence or absence of cysteines at positions corresponding to the conserved disulfide-bonding sites. Predicted N-terminal–ECL2 disulfide-bonding partners based on AlphaFold structural models are also indicated where applicable.

Table S4 Summary of pEC50 and Emax values for concentration–response experiments.

pEC50 and Emax values derived from individual biological replicates for all concentration–response experiments presented in this study. Dose–response curves were fitted using a three-parameter logistic equation. Values are presented as mean ± S.D. Entries labeled “N/A” indicate datasets for which reliable parameter estimation was not possible because responses did not reach saturation or exhibited excessive variability.

Table S5 pLDDT scores of predicted receptor structures.

Predicted Local Distance Difference Test (pLDDT) scores for AlphaFold2 and AlphaFold3 models analyzed in this study. Average pLDDT values are shown for the N-terminal region, extracellular loop 2 (ECL2), and the full-length receptor where applicable. These scores reflect model confidence and are provided as supplementary structural information; they should not be interpreted as direct measures of receptor stability.

Table S1-S5 were uploaded separately.

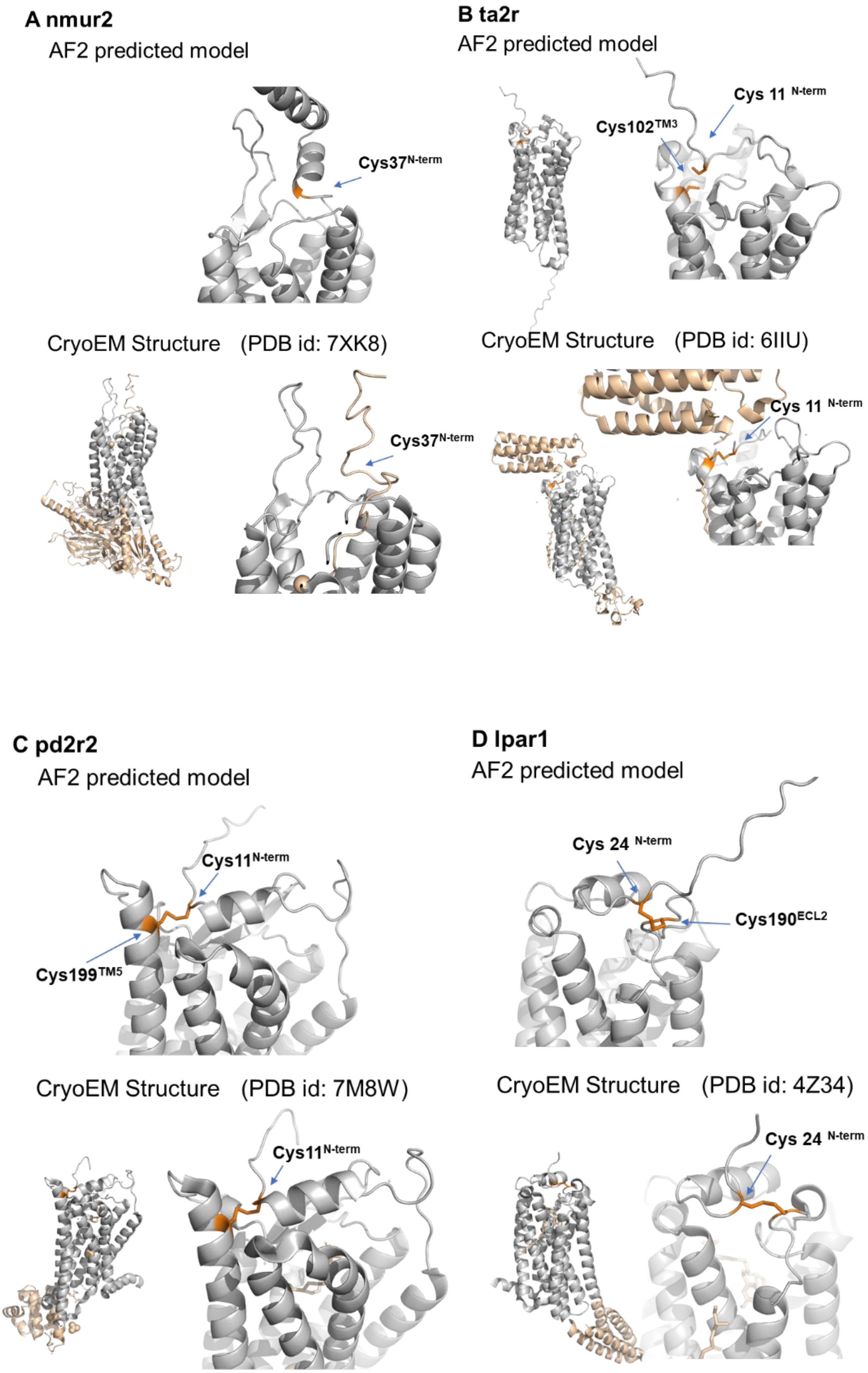

A nmur2
AF2 predicted model
Cys37N-term
CryoEM Structure (PDB id: 7XK8)
Cys37N-term
B ta2r
AF2 predicted model
Cys 11 N-term
Cys102TM3
CryoEM Structure (PDB id: 6IIU)
Cys 11 N-term
C pd2r2
AF2 predicted model
Cys11N-term
Cys199TM5
CryoEM Structure (PDB id: 7M8W)
Cys11N-term
D lpar1
AF2 predicted model
Cys 24 N-term
Cys190ECL2
CryoEM Structure (PDB id: 4Z34)
Cys 24 N-term

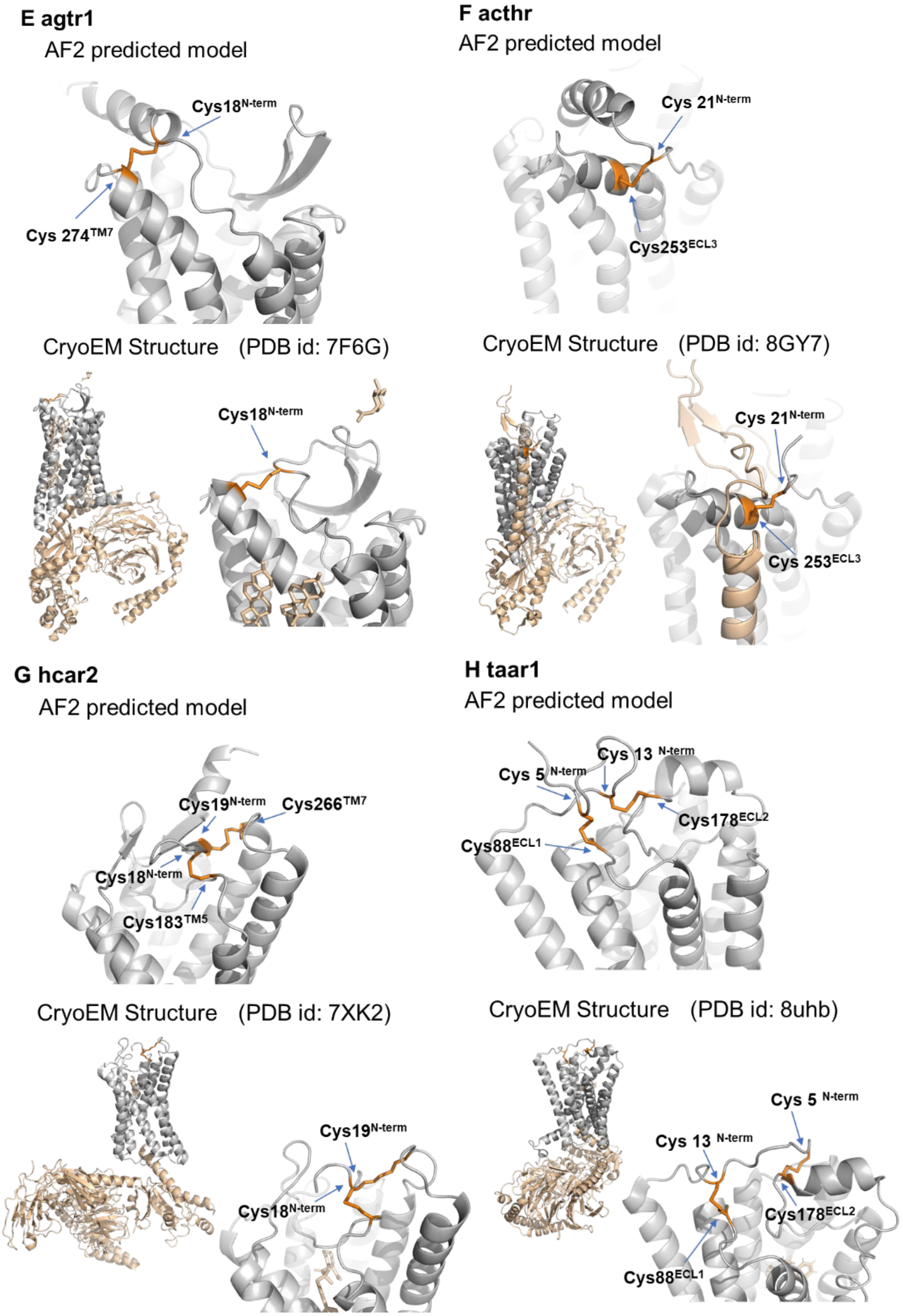

Figure S1 Comparison of the model structure constructed by Alphafold2 of the Human Class A GPCR with the three-dimensional structure of CryoEM, focusing on the intramolecular disulfide bond of the N-terminal cysteine. A:

Neuromedin U Receptor 2(nmur2), B: Thromboxane A2 Receptor(ta2r), C Prostaglandin D2 Receptor 2(pd2r2), D Lysophosphatidic Acid Receptor 1(lpar1), E Angiotensin II Receptor Type 1(agtr1), F Melanocortin 2 Receptor(acthr), G Hydroxycarboxylic Acid Receptor 1(hcar1), H trace amine-associated receptor 1(taar1)

A: Growth hormone-releasing hormone receptor **AF2 model (AF-Q02643-F1-v6)**

B: Glucagon receptor **AF2 model (AF-P47871-F1-v6)**

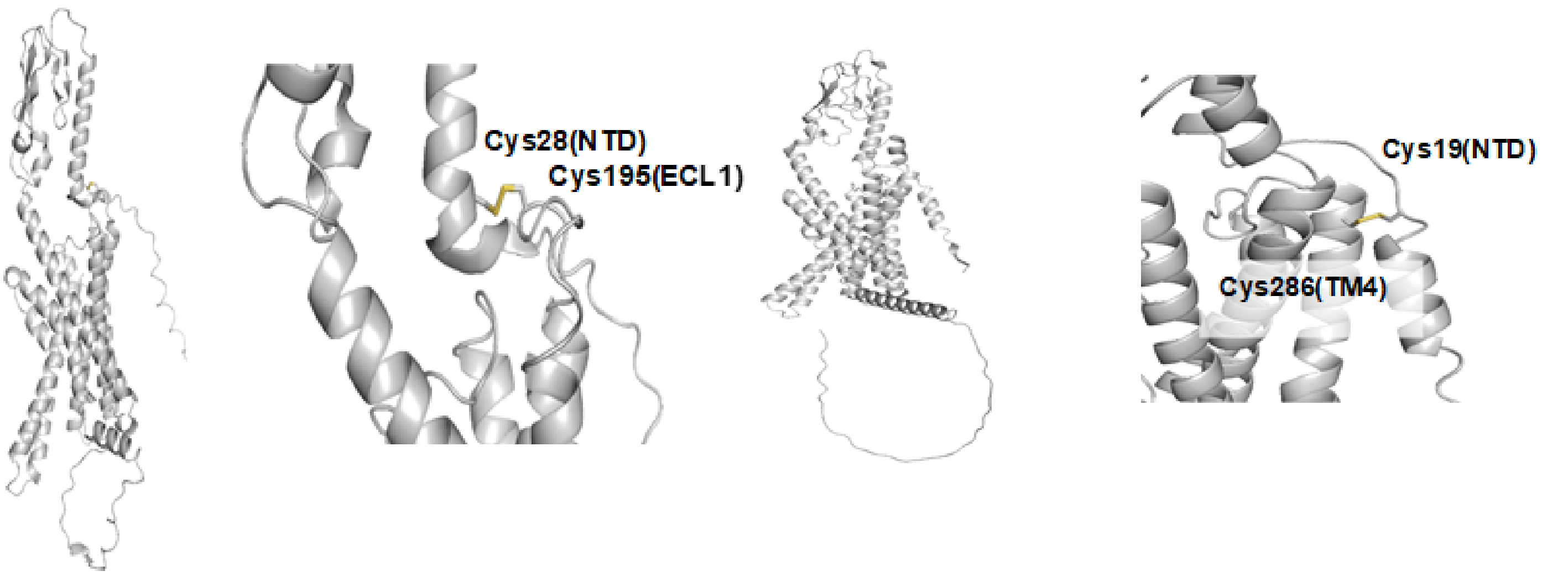

Figure S2 The model structure constructed by Alphafold2 of the Human Class B GPCR focusing on the intramolecular disulfide bond of the cysteine on N-terminal domain. A: Growth hormone-releasing hormone receptor, B: Glucagon receptor

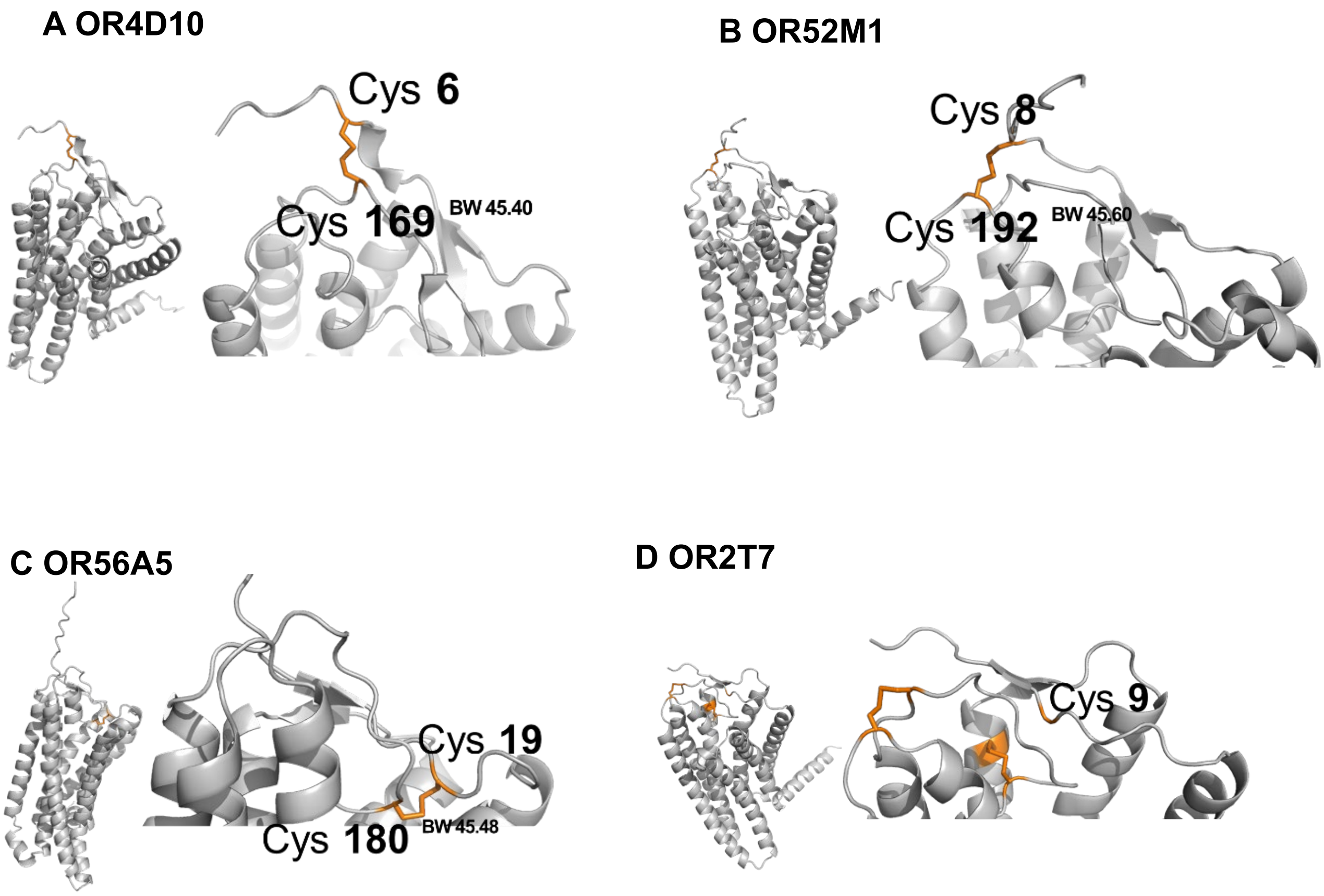

Figure S3 Predicted structures of Human ORs which have an intramolecular disulfide bond using Alphafold2 A: OR4D10, B:OR52M1, C: OR56A5, D: OR2T7

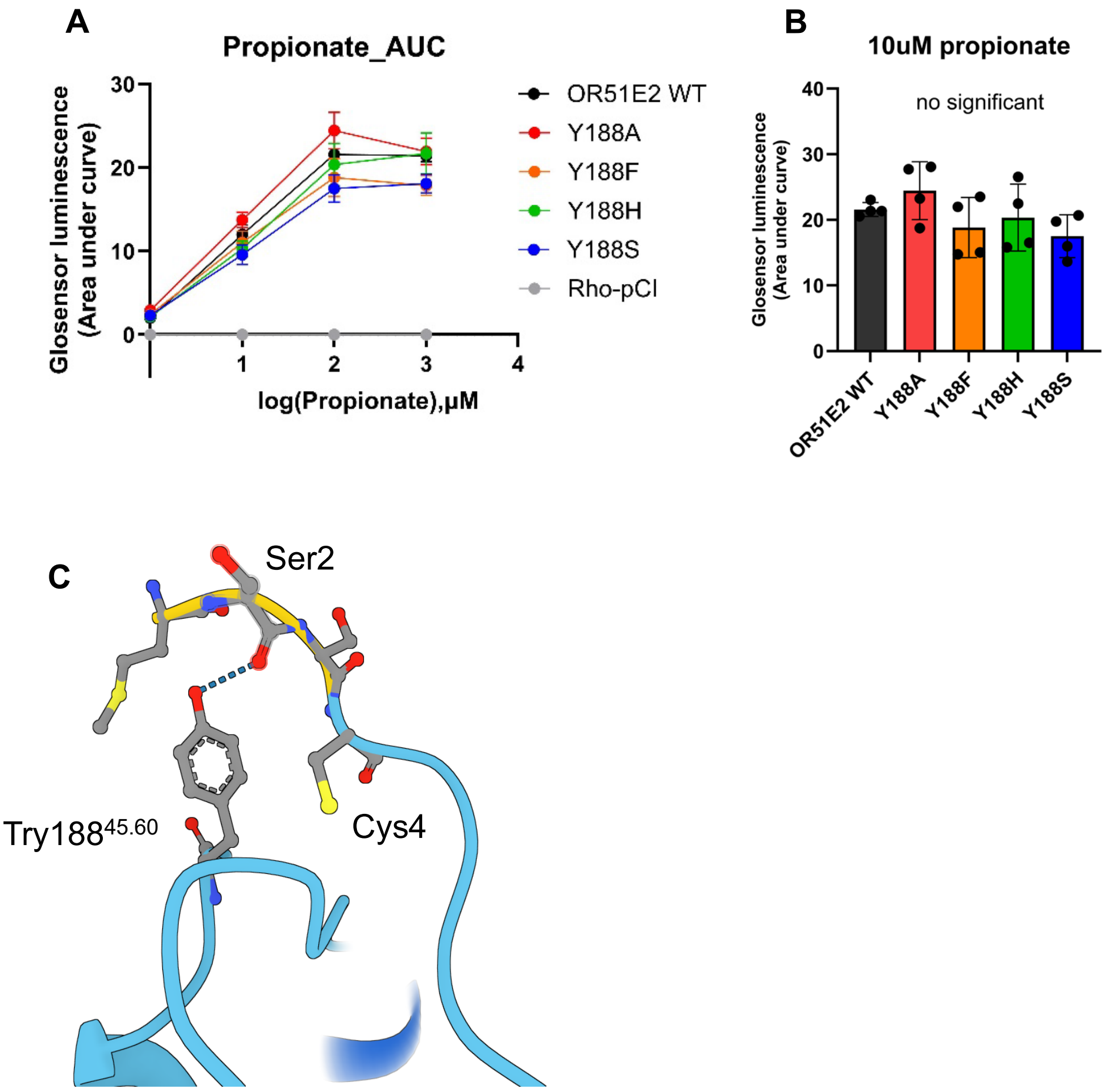

Figure S4 Effect of Tyr188$^{45.60}$ substitution in human OR51E2

(A) Dose–response analysis of OR51E2 wild type (WT; black), Y188A (red), Y188F (orange), Y188H (green), Y188S (blue), and Rho-pCI vector control (gray) in response to propionate. Error bars represent standard deviation (S.D.) with n = 3. Statistical analysis was performed using one-way ANOVA followed by Dunnett's test (no significant differences). (B) Comparison of the area under the curve (AUC) derived from dose–response profiles at 10 μM propionate for each mutant shown in (A). (C) Enlarged view of the N-terminal region in the structural model of OR51E2. Tyr188$^{45.60}$ is predicted to form a hydrogen bond with Ser2 in the N-terminus.

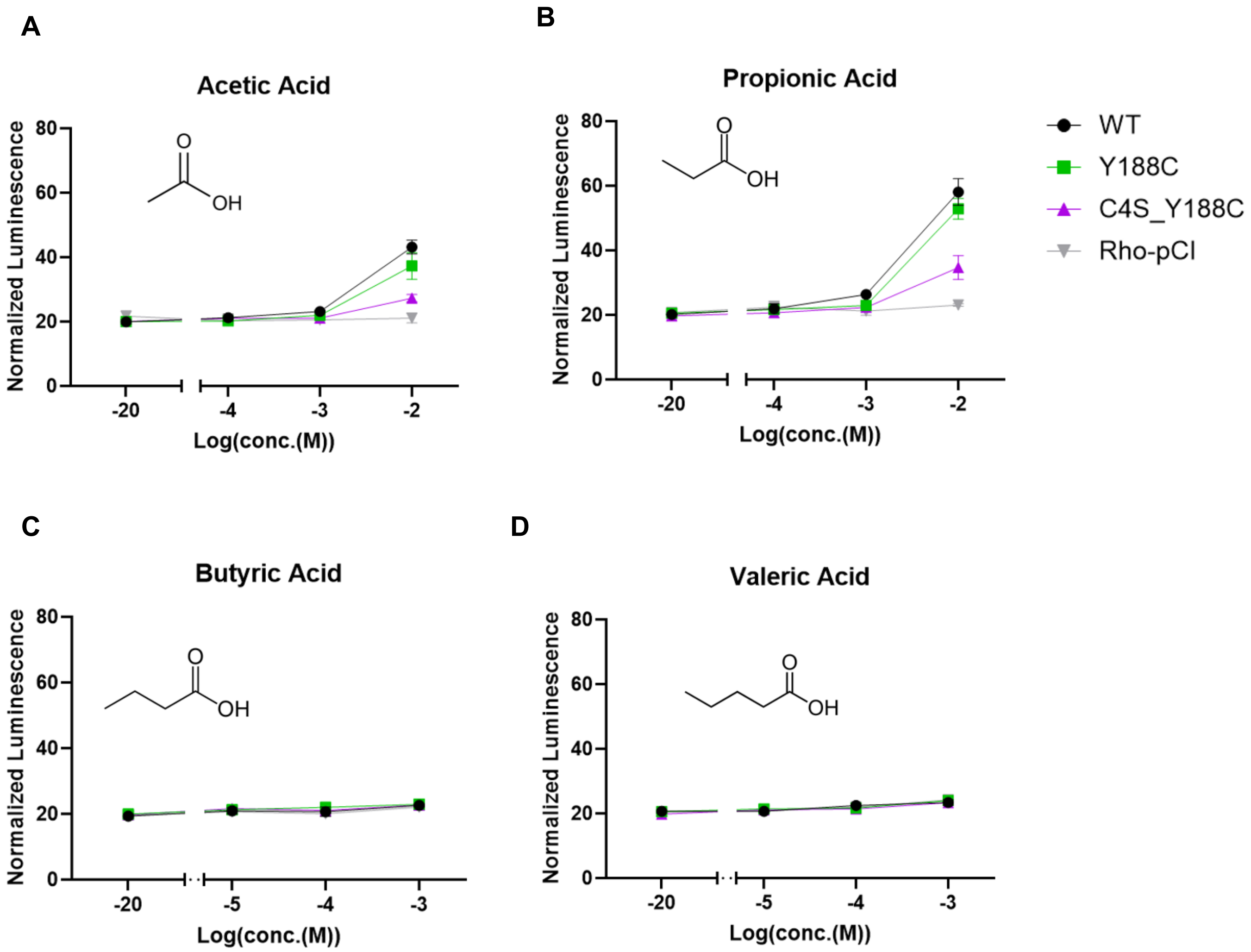

Figure S5 Ligand selectivity of wild-type OR51E2 and disulfide-bond mutants toward short-chain carboxylic acids. To investigate whether alternative disulfide-bond configurations influence ligand selectivity, the responses of wild-type (WT) OR51E2 and the indicated mutants were evaluated against a series of carboxylic acids with different carbon chain lengths. Responses of WT OR51E2, Y188C mutant, and C4S+Y188C mutant to acetic acid (A), propionic acid (B), butyric acid (C), and valeric acid (D).

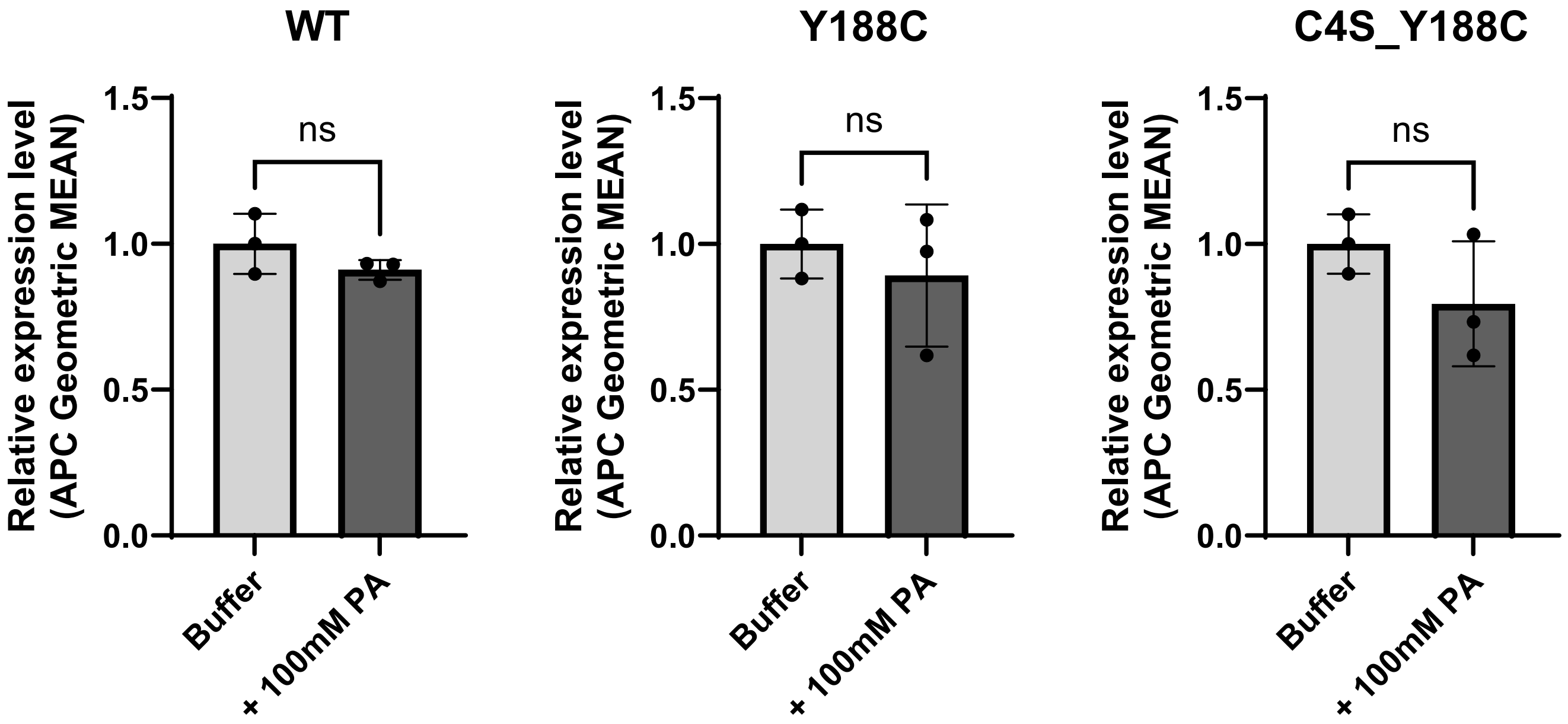

Figure S6 Analysis of internalization of OR51E2 wild-type and mutants

We investigated whether the receptors are internalized inside the cell when exposed to ligand solution. The vertical axes show the cell surface expression of each odorant 2 hours after exposed to buffer without ligand or the 100mM propionic acid solution.

The lower expression level means the higher possibility that the receptors are internalized inside cells.

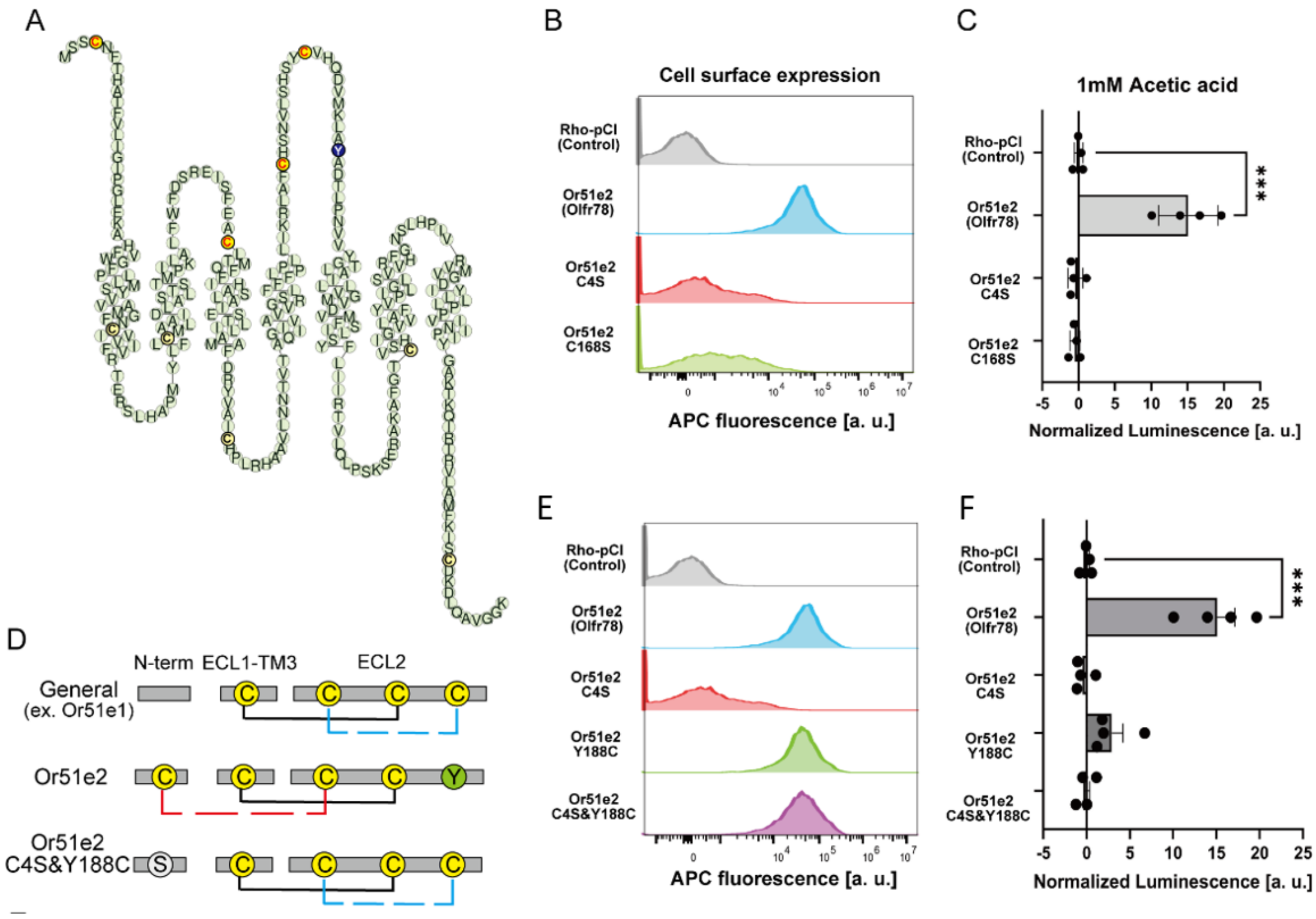

Figure S7 Importance of the disulfide bond between Cys4$^{N\text{-}term}$ and Cys168$^{45.40}$ in Mouse Or51e2

A) Snake plot showing the amino acid sequence of Or51e2 with yellow circles indicating the Cysteins, and a blue circle indicating Tyr188. Yellow circles with red character indicates the Cysteines composing the disulfide bond linking N-terminus, ECL1, ECL2 domain. B) Cell surface expression of Or51e2 WT(cyan), C4S(red), C168S(green), and Rho-pCI vector control (gray) transfected in HEK293T cells, measured and represented on X-axis as APC fluorescence and on the Y-axis as cell number. A total of 10,000 cells were analyzed with gating permitting GFP positive, single, spherical and viable cells. C) The comparison of figures representing the area under curve of the dose response of Or51e2 WT, C4S, C168S, and Rho-pCI vector control to 1mM acetic acid. Error bars represent standard deviation (S.D.) with n=4. Multiple comparisons were performed using one-way ANOVA followed by Dunnett's test (***$p<0.001$) for each concentration. D) Figures showing the location of Cysteines forming a intramolecular disulfide bond in General ORs without N-terminal Cysteine (upper). Or51e2 (middle), Or51e2 C4S,Y188C (lower). E) Cell surface expression of Or51e2 WT(cyan), C4S(red), Y188C(green), C4S,Y188C(purple), and Rho-pCI vector control (gray) transfected in HEK293T cells, measured and represented on X-axis as APC fluorescence and on the Y-axis as cell number. A total of 10,000 cells were analyzed with gating permitting GFP positive, single, spherical and viable cells. F) The comparison of figures representing the area under curve of the dose response of Or51e2 WT, C4S, Y188C, C4S,Y188C, and Rho-pCI vector control to 1mM acetic acid. Error bars represent standard deviation (S.D.) with n=4. Multiple comparisons were performed using one-way ANOVA followed by Dunnett's test (＊＊＊$p<0.001$) for each concentration.

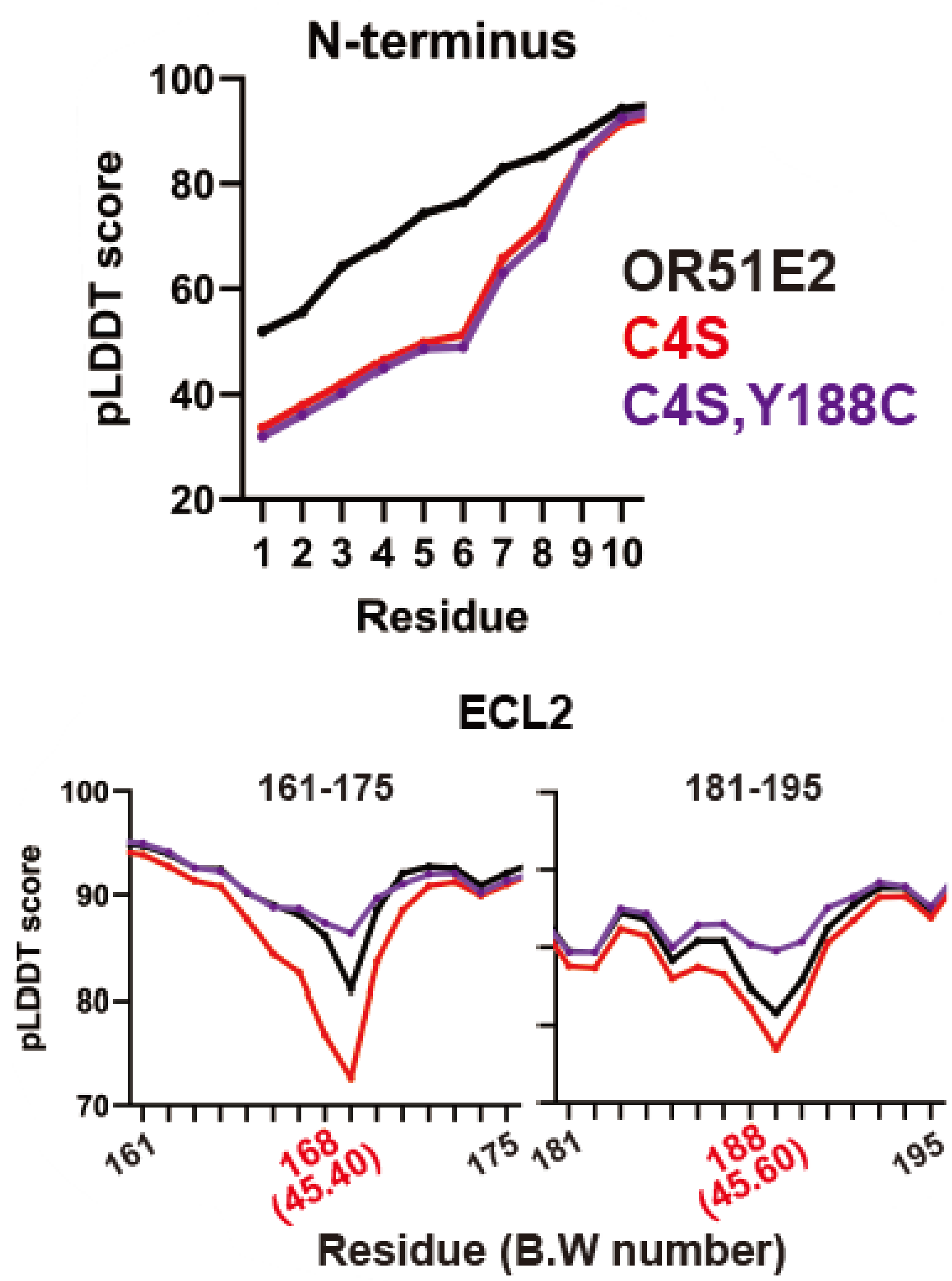

Figure S8 Predicted Local Distance Difference Test (PLDDT) scores of each residue in OR51E2 WT (black), C4S (red), and C4S+Y188C (purple).

X-axis: residue position; Y-axis: PLDDT score. Top: scores of the N-terminal 10 residues; bottom: scores of 30 residues in ECL2. Calculated using ColabFold v1.5.5: AlphaFold2 with MMseqs2. Each dot represents one residue's score.

A OR51E2 WT

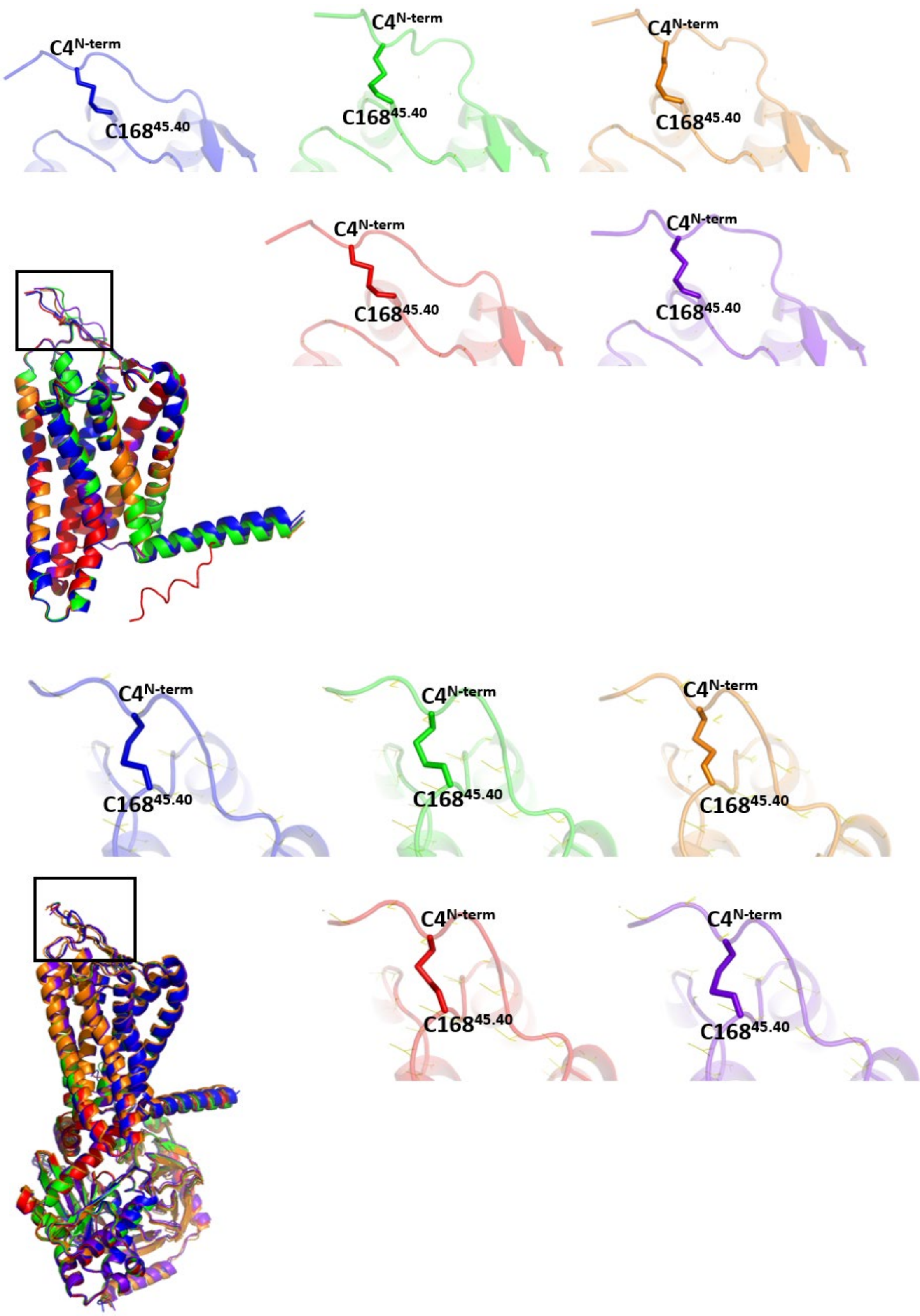

C4N-term
C16845.40
C4N-term
C16845.40
C4N-term
C16845.40
C4N-term
C16845.40
C4N-term
C16845.40
C4N-term
C16845.40
C4N-term
C16845.40
C4N-term
C16845.40
C4N-term
C16845.40
C4N-term
C16845.40

B OR51E2   C4S, Y188C

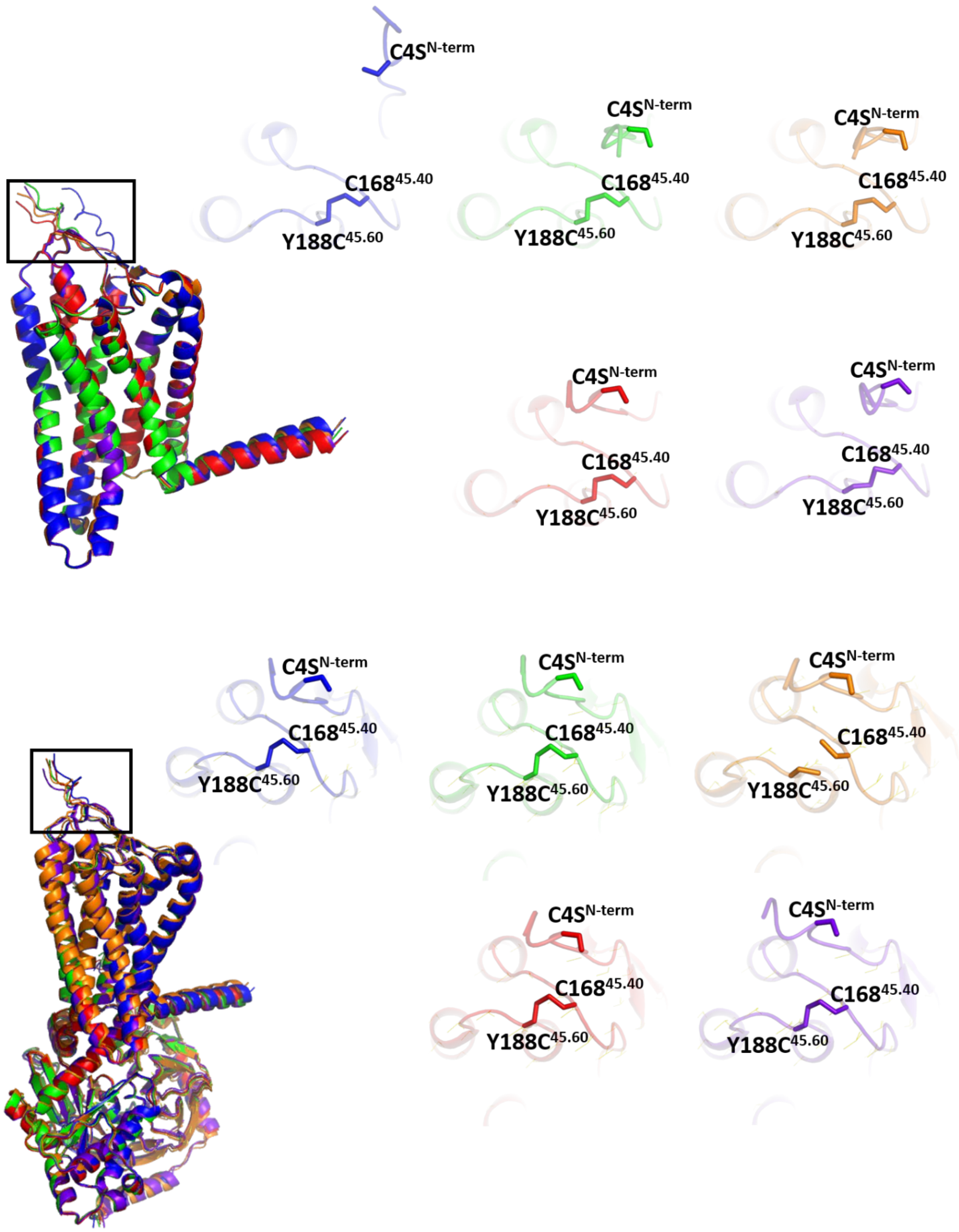

C OR51E2 Y188C

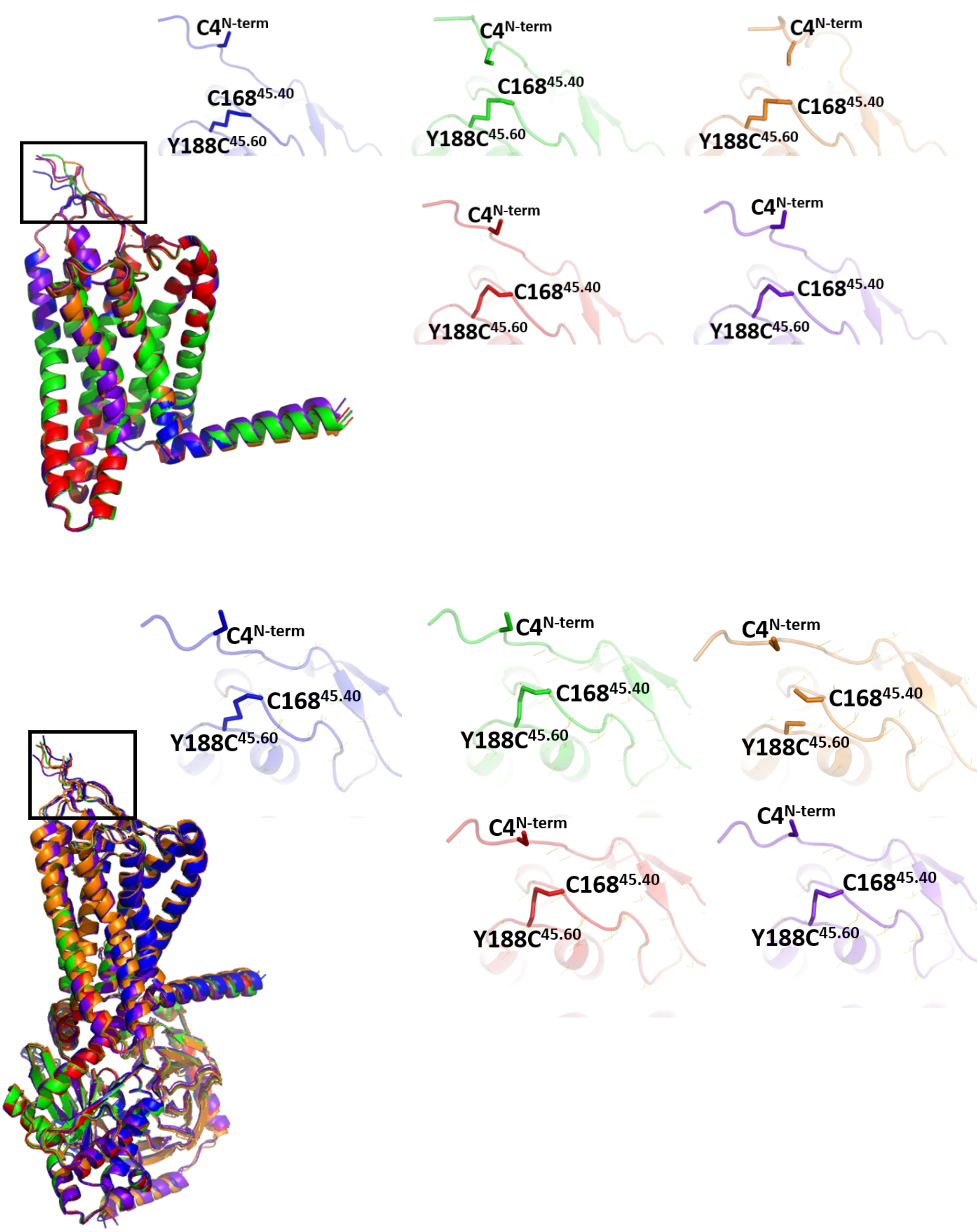

D OR51E2 S2C, C4S

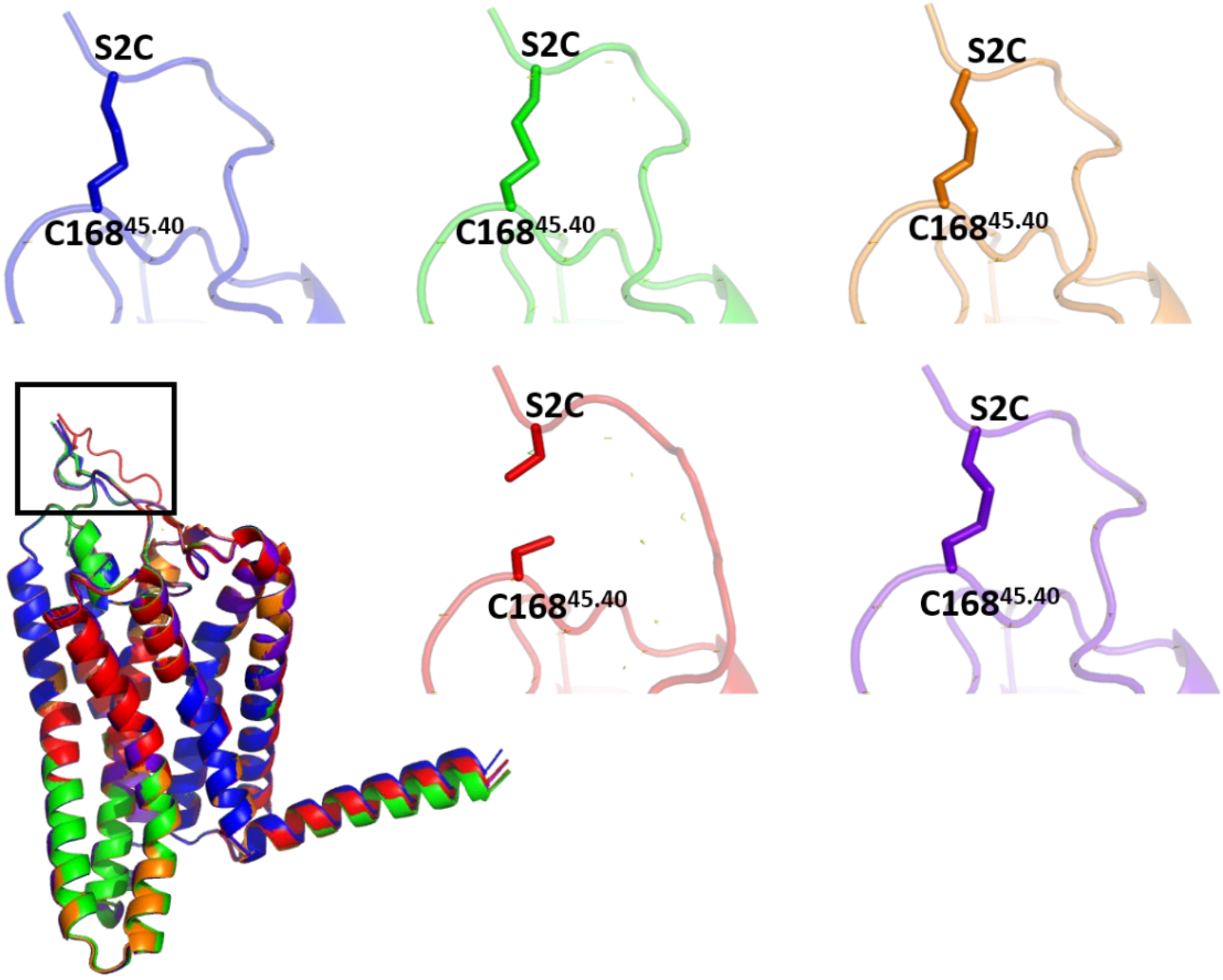

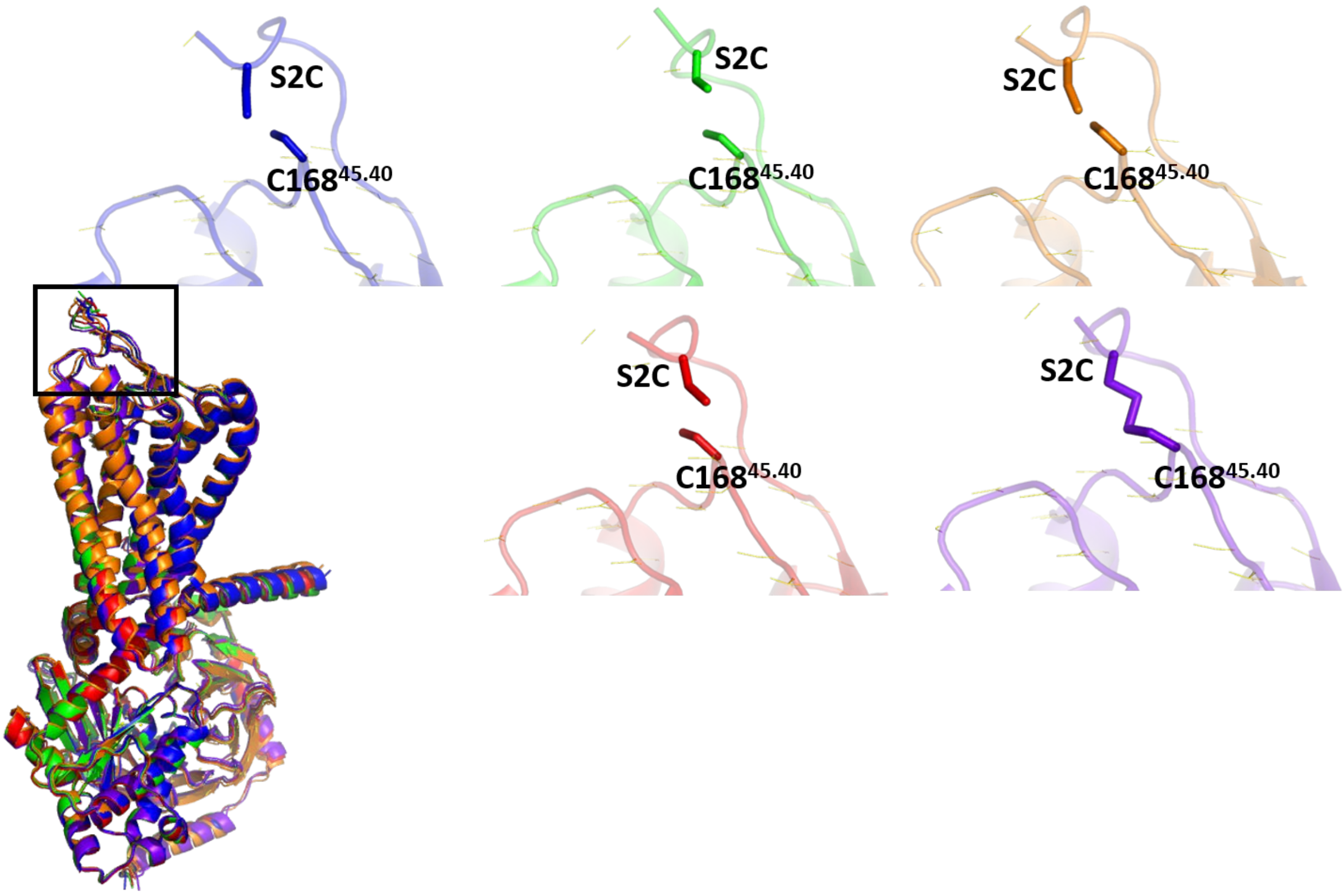

E OR51E2 S3C, C4S

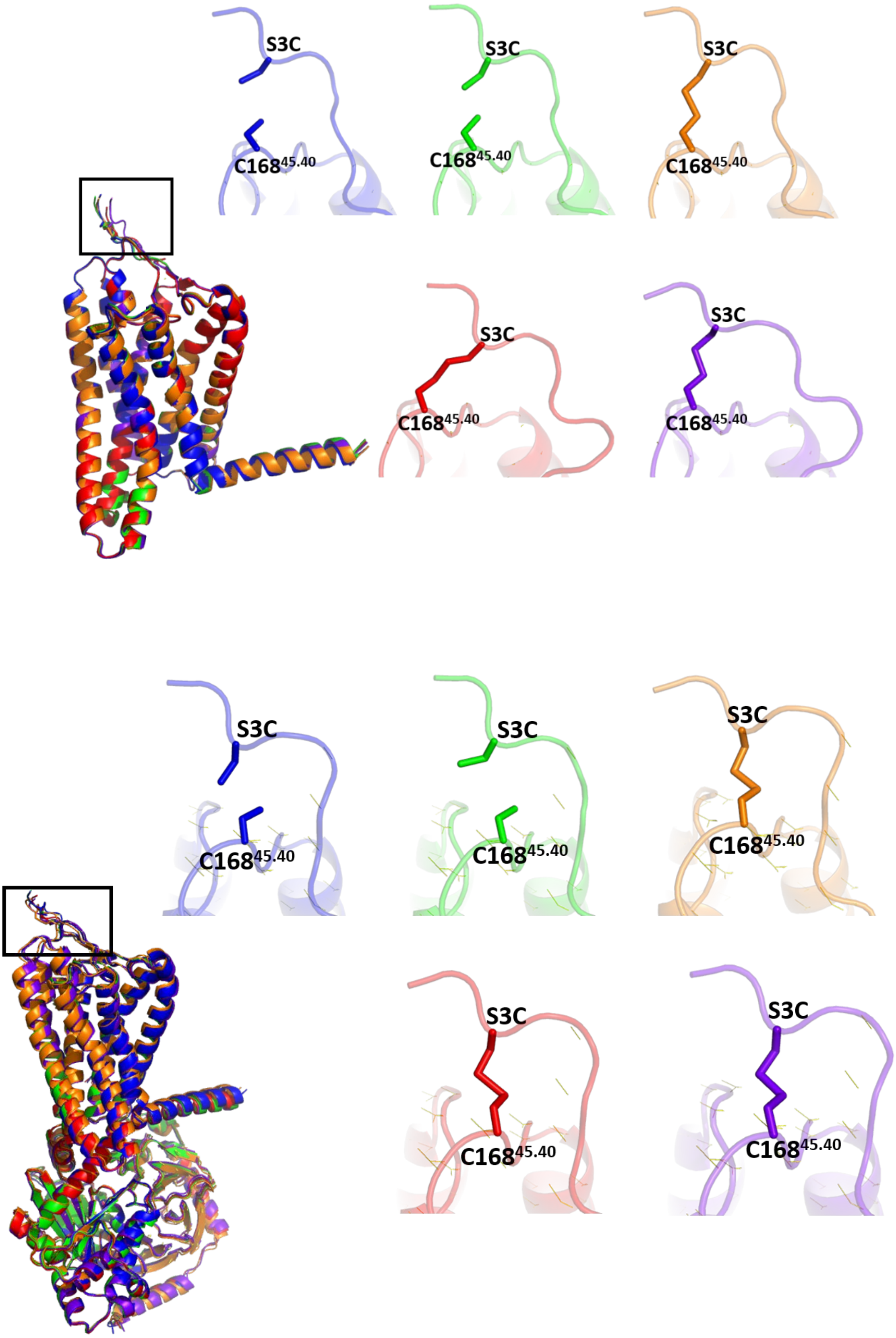

F OR51E2 C4S, N5C

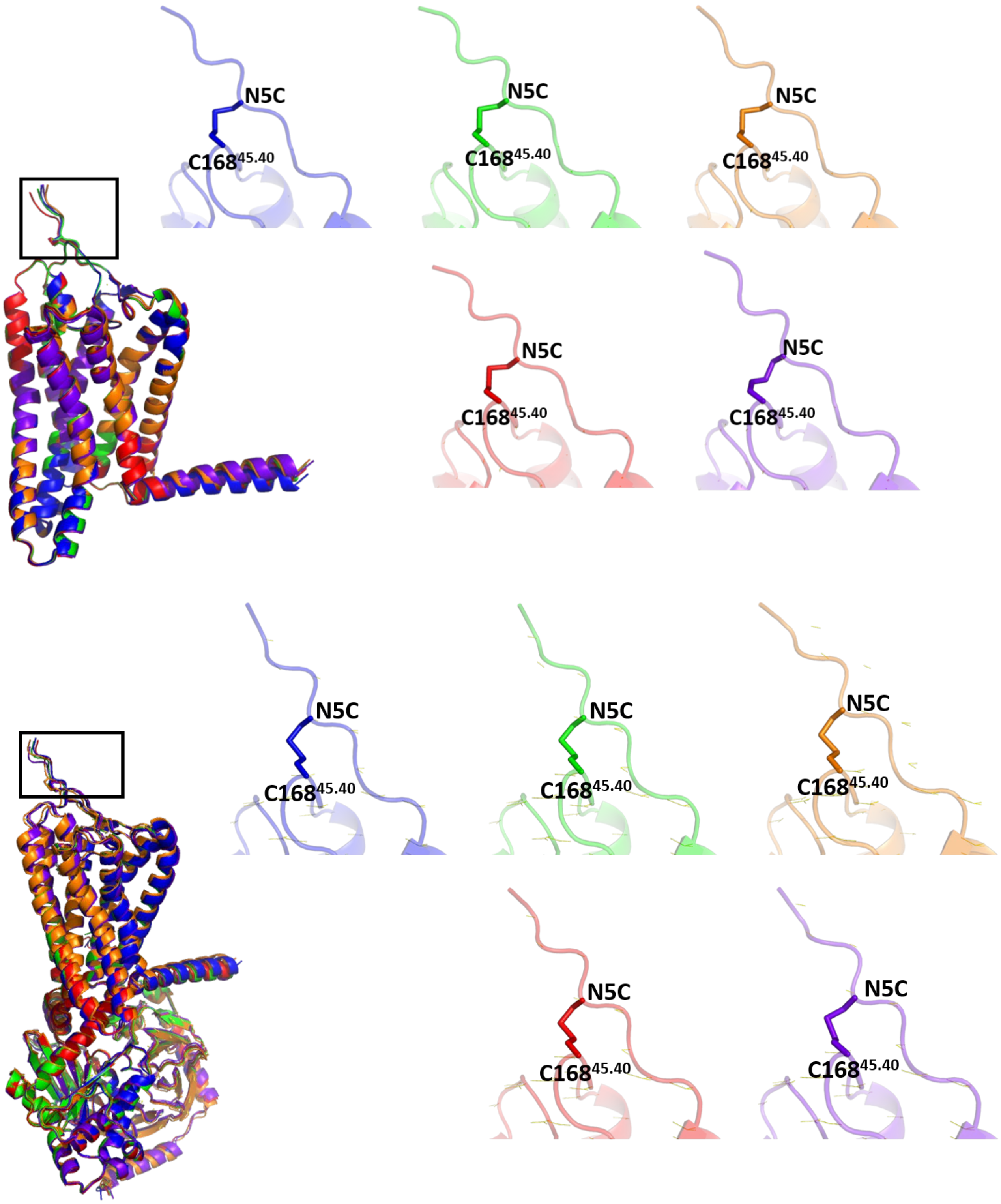

G OR51E2 C4S, F6C

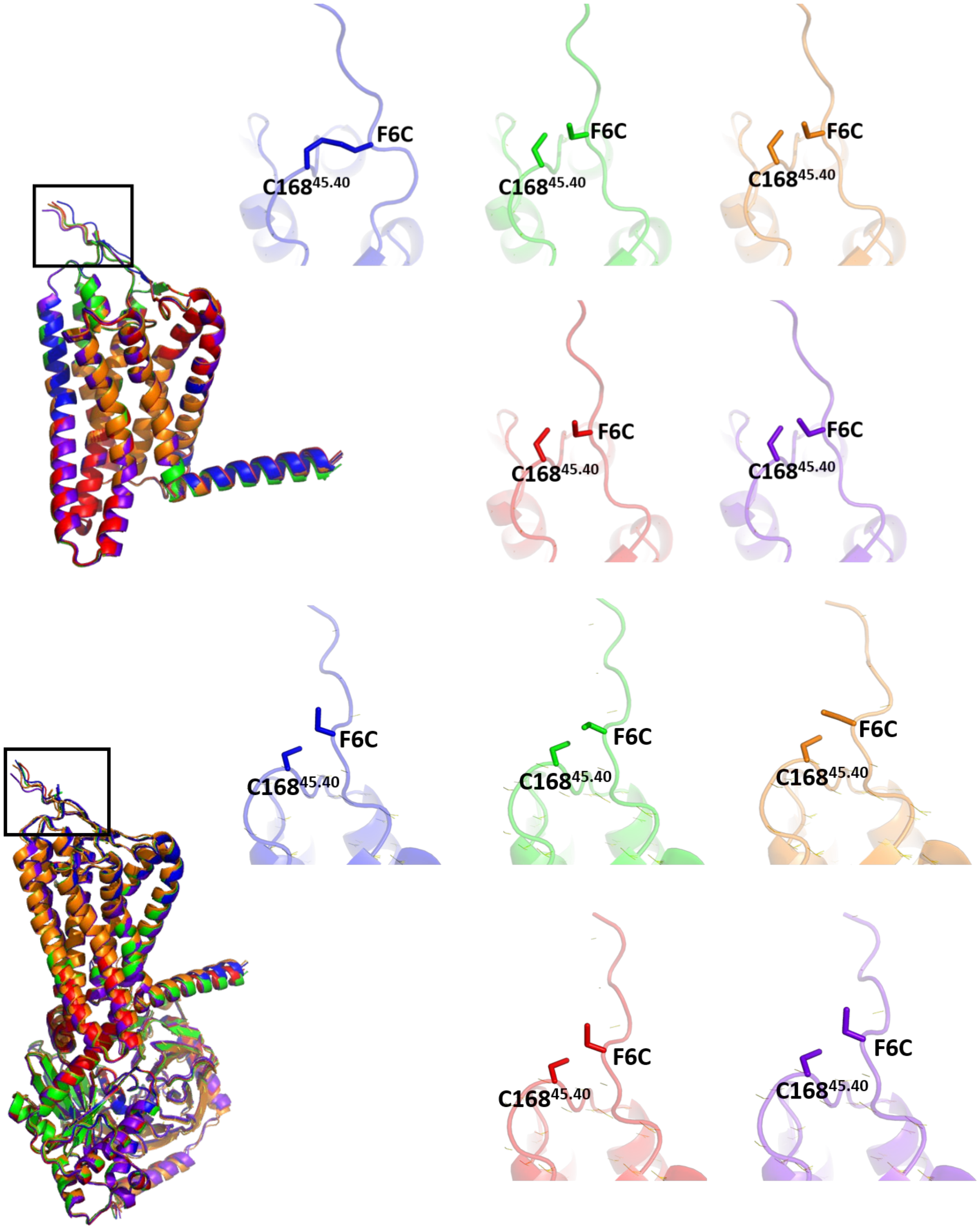

H OR51E2 C4S, T7C

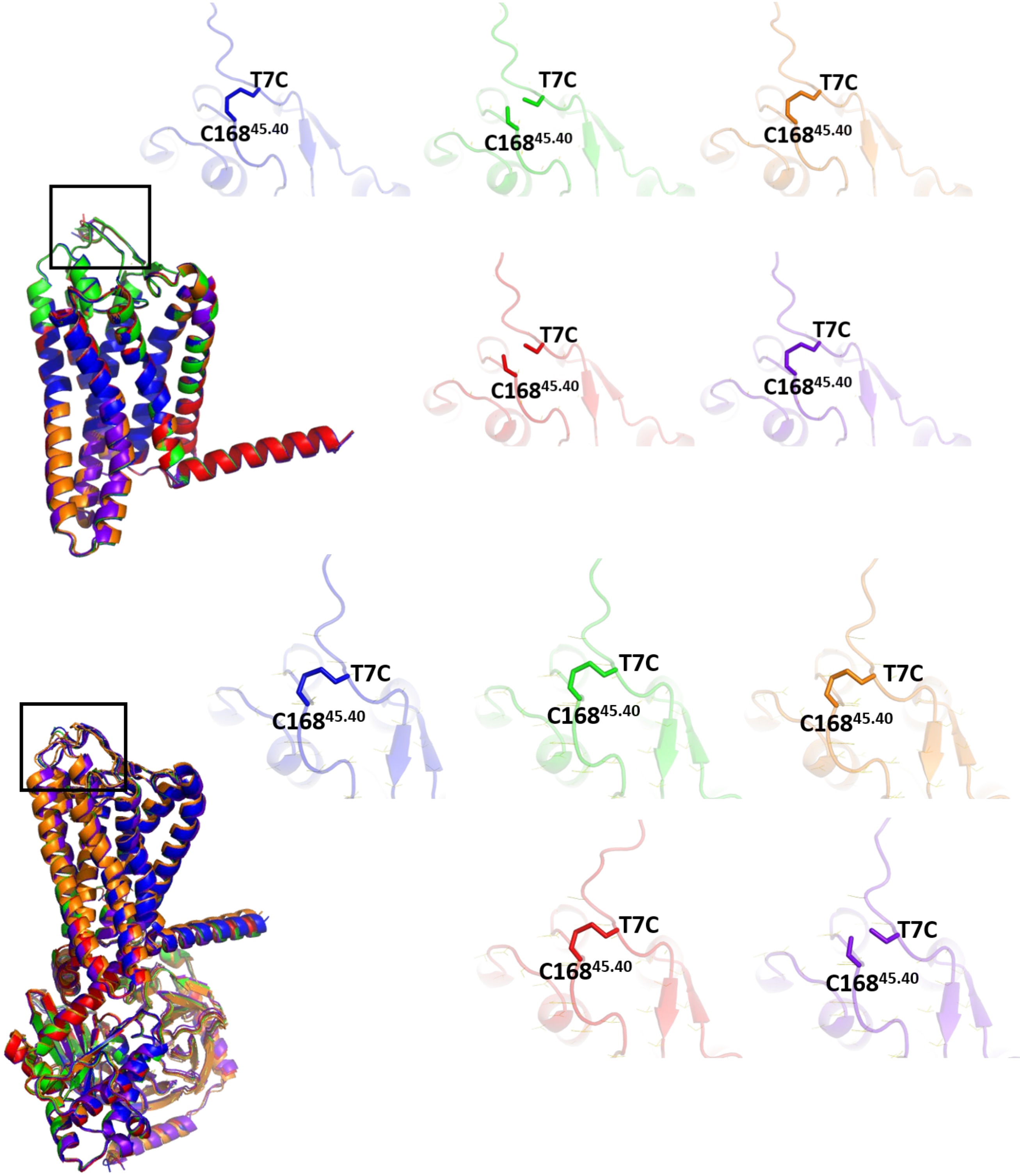

I OR51E2　C4S, H8C

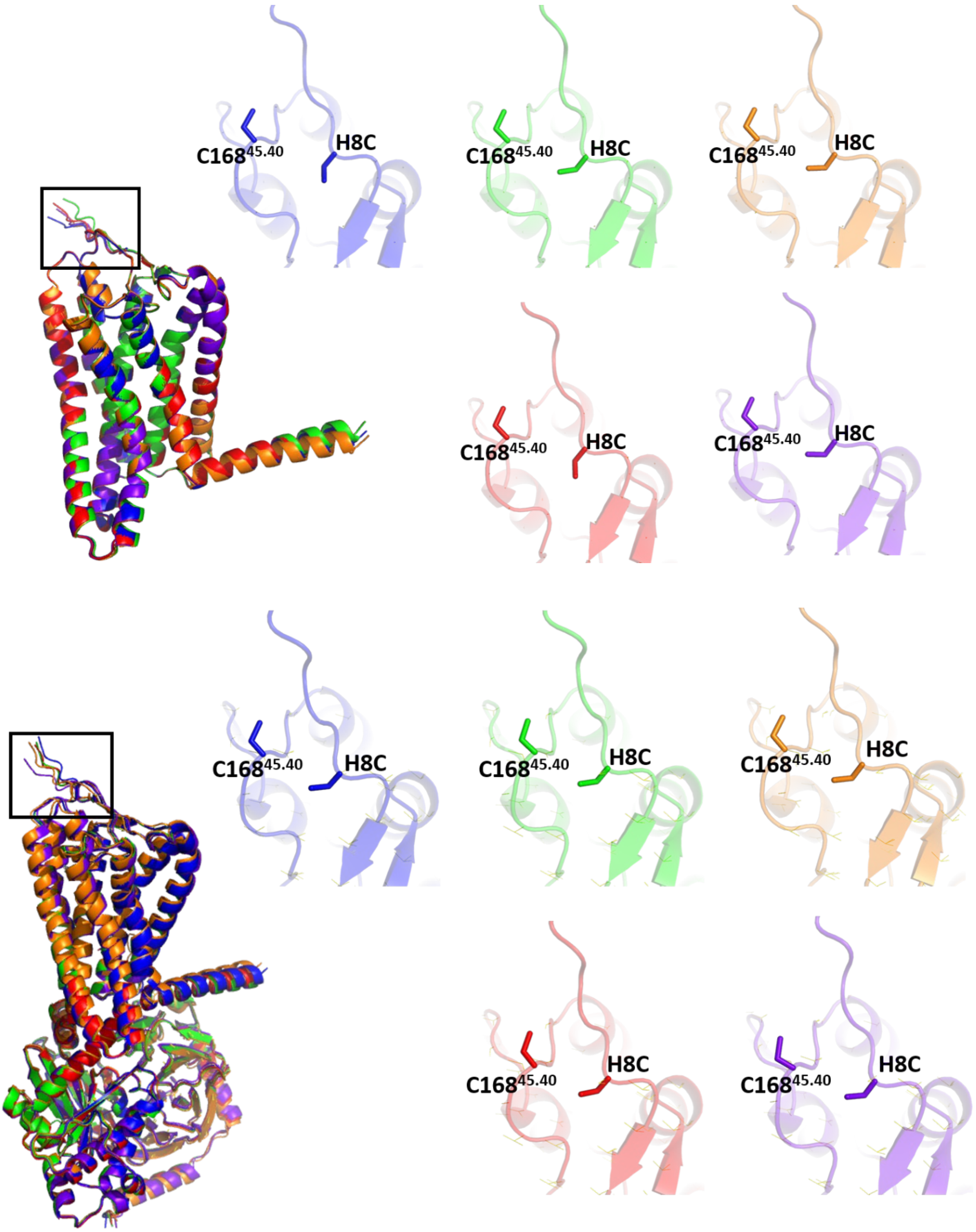

Figure S9 Structural alignment of 5 structures generated by AlphaFold3 as for human OR51E2 wild-type and mutants.

Each figure shows the overlay structure of the only receptor in upper area, and the overlay structures of the receptor in complex with the Golf heterotrimer and propionic acid in the lower area.

Blue: model0, Green: model1, Orange: model2, Red: model3, Purple: model4

The area of black box includes the N-terminal and ECL2 domain, and enlarged views of the area are shown on the right.

A

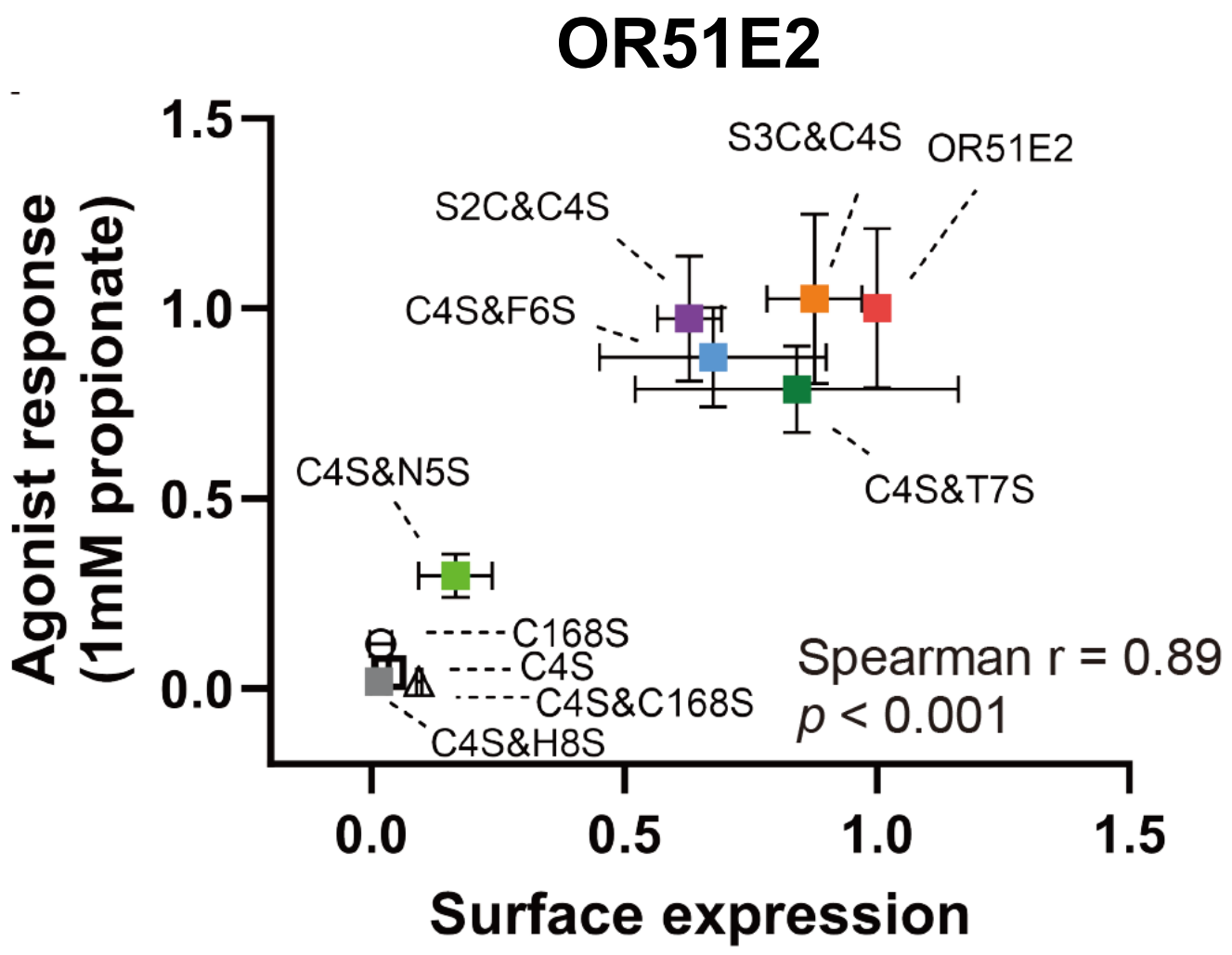

B

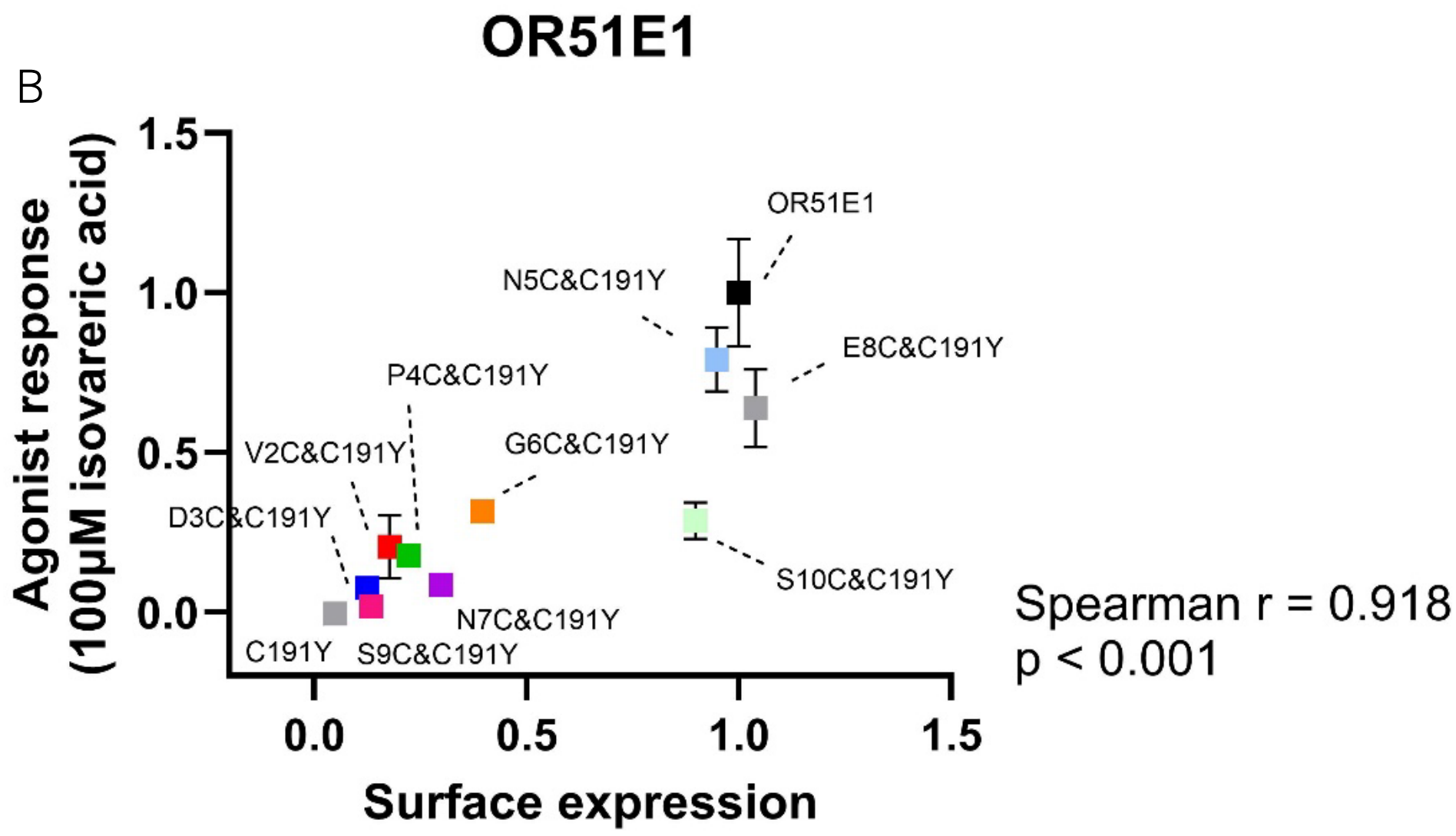

Figure S10 Correlation analysis between cell surface expression and agonist response.

(A) OR51E2 and its mutants. (B) OR51E1 and its mutants.

A OR51E2 wild-type and mutants pLDDT score

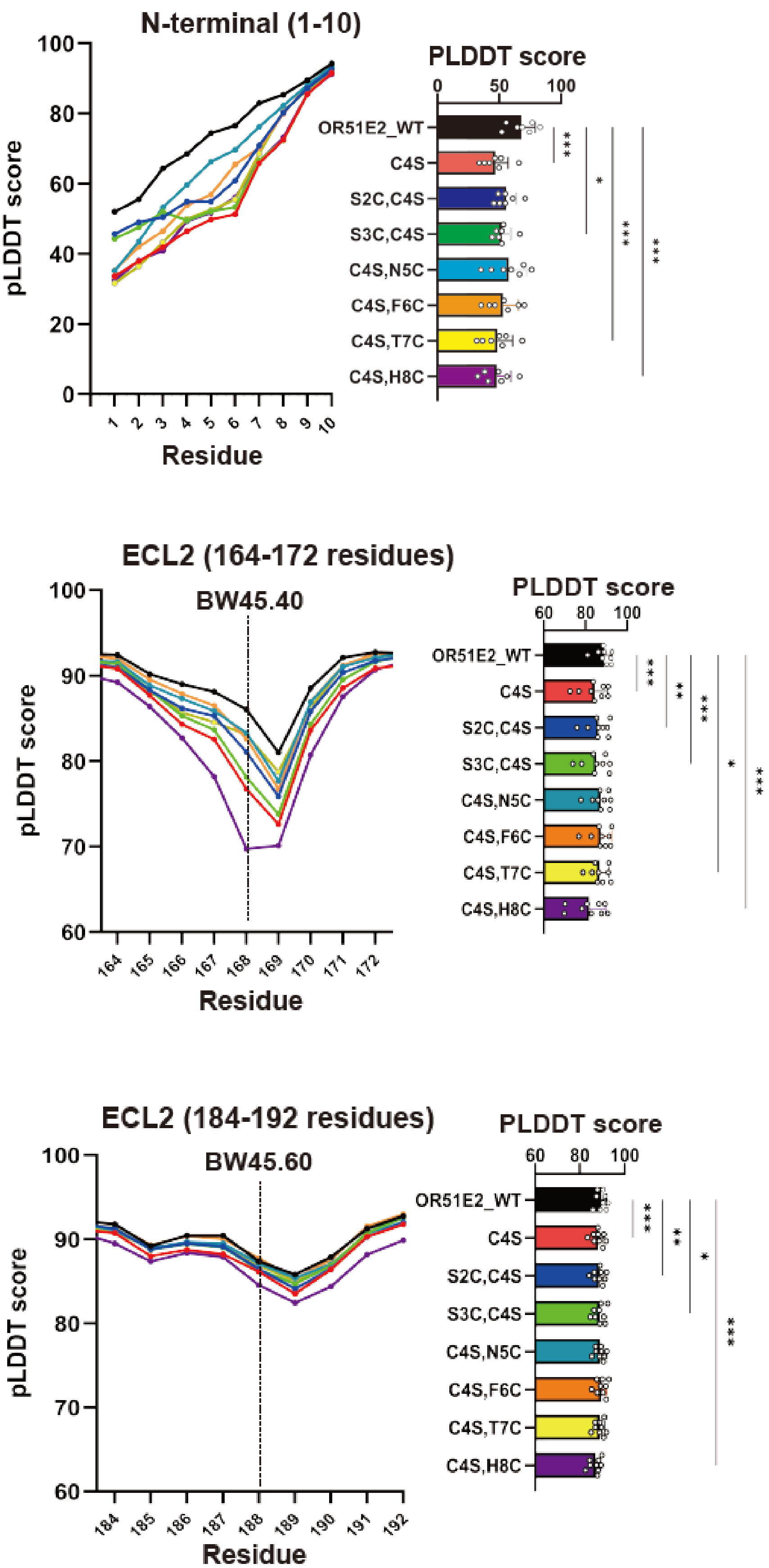

B OR51E1 wild-type and mutants pLDDT score

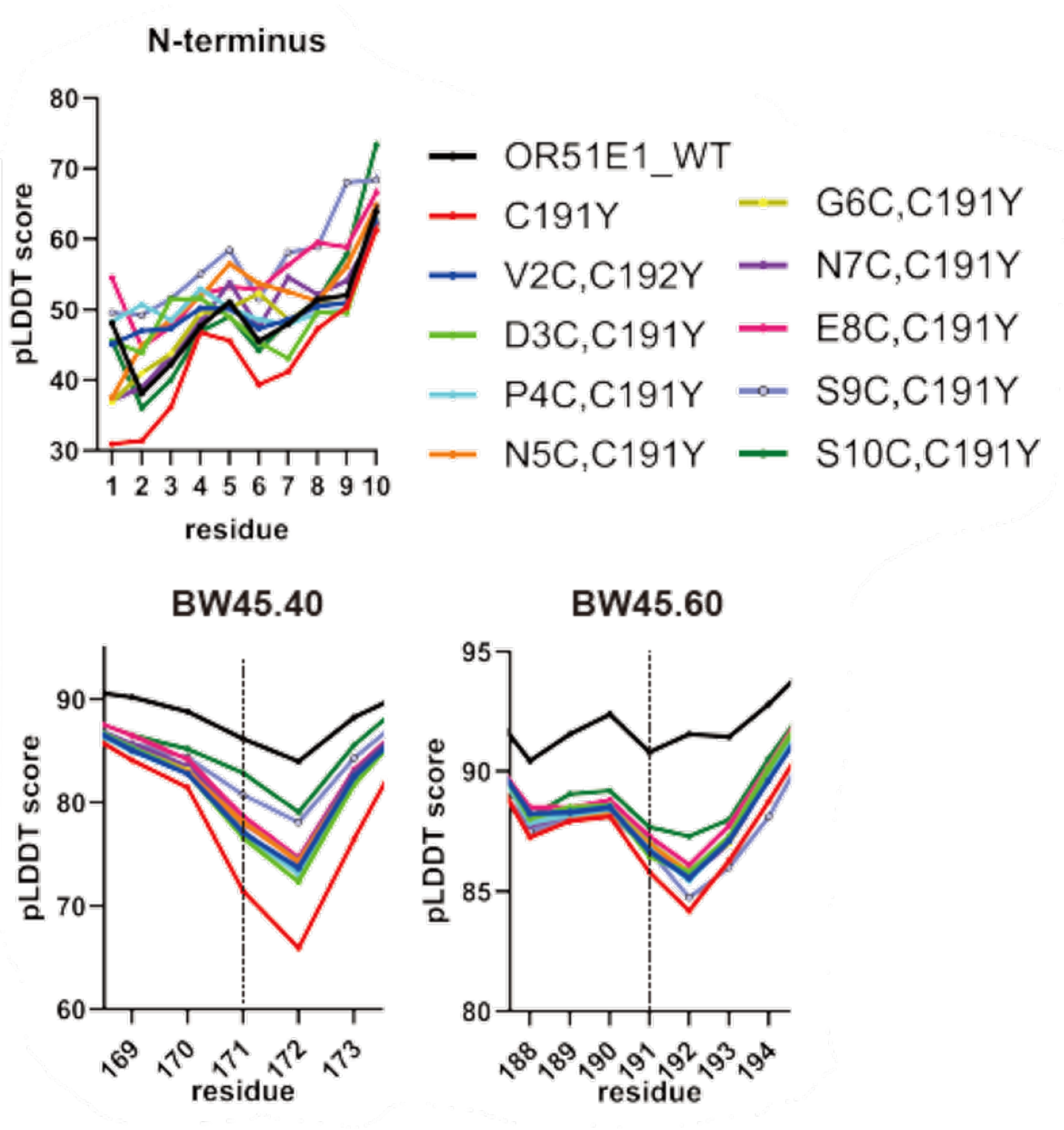

C Or8w1 wild-type and mutants pLDDT score

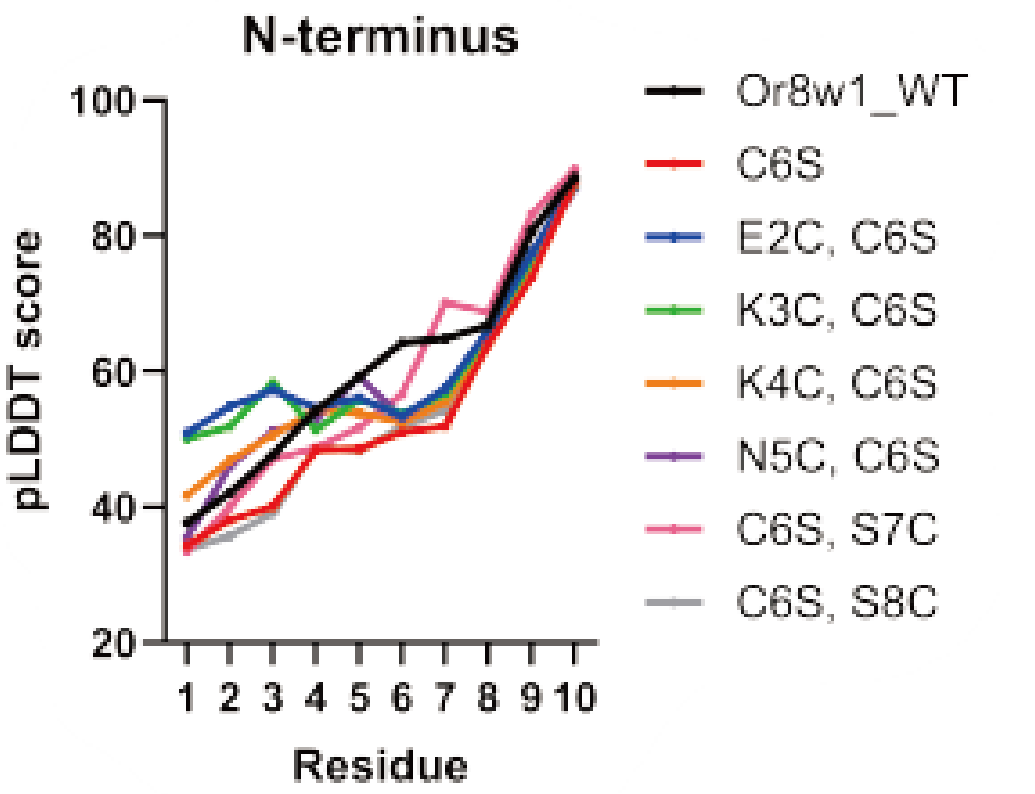

Figure S11 Predicted structural stability (pLDDT scores) of each mutant.

pLDDT values were calculated using ColabFold. Statistical significance of differences from the wild type was evaluated by Dunnett's test (*$p < 0.05$, ** $p < 0.01$, *** $p < 0.001$).

A) the upper panel: scores for the N-terminal 10 residues; the middle and lower panel: scores for residues surrounding BW45.40 and BW45.60 on ECL2, respectively. Left graphs display individual residue scores, while right graphs summarize regional averages.

B) predicted local structural stability (pLDDT scores) of OR51E1 WT (black), C191Y (red), and each double mutant. The upper graph shows scores for the N-terminal 10 residues. Lower graphs show scores for 9 residues surrounding BW45.40 (left) and BW45.60 (right) on ECL2.

C) PLDDT scores for the N-terminal 10 residues of mutants shown in panel E. Each dot represents one residue.
